# Systematic identification of genes with a cancer-testis expression pattern in 19 cancer types

**DOI:** 10.1038/ncomms10499

**Published:** 2016-01-27

**Authors:** Cheng Wang, Yayun Gu, Kai Zhang, Kaipeng Xie, Meng Zhu, Ningbin Dai, Yue Jiang, Xuejiang Guo, Mingxi Liu, Juncheng Dai, Linxiang Wu, Guangfu Jin, Hongxia Ma, Tao Jiang, Rong Yin, Yankai Xia, Li Liu, Shouyu Wang, Bin Shen, Ran Huo, Qianghu Wang, Lin Xu, Liuqing Yang, Xingxu Huang, Hongbing Shen, Jiahao Sha, Zhibin Hu

**Affiliations:** 1 State Key Laboratory of Reproductive Medicine, Nanjing Medical University, Nanjing 210029, China; 2 Department of Epidemiology and Biostatistics, Jiangsu Key Lab of Cancer Biomarkers, Prevention and Treatment, Collaborative Innovation Center for Cancer Personalized Medicine, School of Public Health, Nanjing Medical University, Nanjing 210029, China; 3 Department of Bioinformatics, School of Basic Medical Sciences, Nanjing Medical University, Nanjing 210029, China; 4 Jiangsu Key Laboratory of Molecular and Translational Cancer Research, Collaborative Innovation Center For Cancer Personalized Medicine, Nanjing Medical University Affiliated Cancer Hospital, Nanjing 210009, China; 5 Department of Molecular Cell Biology and Toxicology, Jiangsu Key Lab of Cancer Biomarkers, Prevention & Treatment, Collaborative Innovation Center For Cancer Personalized Medicine, School of Public Health, Nanjing Medical University, Nanjing 210029, China; 6 Digestive Endoscopy Center, the First Affiliated Hospital of Nanjing Medical University, Nanjing 210029, China; 7 Department of Molecular and Cellular Oncology, Cancer Biology Program, Center for RNA Interference and Non-Coding RNAs, the University of Texas MD Anderson Cancer Center, Houston, Texas 77030, USA; 8 School of Life Science and Technology, Shanghai Tech University, 100 Haike Road, Pudong New Area, Shanghai 201210, China

## Abstract

Cancer-testis (*CT*) genes represent the similarity between the processes of spermatogenesis and tumorigenesis. It is possible that their selective expression pattern can help identify driver genes in cancer. In this study, we integrate transcriptomics data from multiple databases and systematically identify 876 new *CT* genes in 19 cancer types. We explore their relationship with testis-specific regulatory elements. We propose that extremely highly expressed *CT* genes (EECTGs) are potential drivers activated through epigenetic mechanisms. We find mutually exclusive associations between EECTGs and somatic mutations in mutated genes, such as *PIK3CA* in breast cancer. We also provide evidence that promoter demethylation and close non-coding RNAs (namely, CT-ncRNAs) may be two mechanisms to reactivate *EECTG* gene expression. We show that the meiosis-related EECTG (*MEIOB*) and its nearby CT-ncRNA have a role in tumorigenesis in lung adenocarcinoma. Our findings provide methods for identifying epigenetic-driver genes of cancer, which could serve as targets of future cancer therapies.

## Abstract (web summary)

Genes normally expressed in the testis but aberrantly expressed in cancer are termed cancer testis antigens. In this study, the authors catalogue the expression of these genes in 19 different cancer types and correlate expression with some somatically mutated oncogenes.

## Graphical abstract

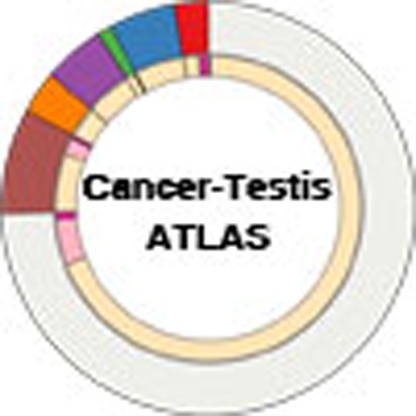

Over the past decade, genome-wide analyses via exome sequencing have become routine in cancer research. Large cooperative projects such as The Cancer Genome Atlas (TCGA), as well as other independent studies, have identified hundreds of genes that may promote tumorigenesis when altered by intragenic mutations^1^,^2^,^3^. These findings dramatically expand the range of targetable alterations and ultimately advance personalized therapy. However, individuals with the same tumour type are highly heterogeneous and contain diverse genomic alterations^4^,^5^. Studies suggest that only a few mutations in these mutation-driver (*mut-driver*) genes are sufficient to trigger cancer^1^,^3^,^6^. The remaining mutations are passengers that confer no selective growth advantage. The majority of somatic point mutations present with low population frequencies^1^,^2^,^7^,^8^,^9^,^10^,^11^, with only a handful of mutations observed in >5% of patients. In 12 major cancers investigated in the TCGA project^1^, only 10 significantly mutated genes (SMGs) contain point mutations in >5% of samples. Thus, few cancers can be completely explained by known driver mutations.

Epigenetic alterations, which lead to aberrant expression patterns, are now acknowledged as a universal feature of tumorigenesis. In turn, aberrant expression patterns are an important characteristic of epigenetic-drivers (epi-drivers)^3^. In the present study, we observed a group of genes in which expression was restricted to germ cells and often reactivated and aberrantly expressed in tumour cells. These gene products were named cancer/testis (CT) antigens in initial studies due to their immunogenicity^12^,^13^. The existence of these genes reflects the similarities between the processes of gametogenesis and tumorigenesis. Study of these genes provides valuable understanding of tumorigenesis^12^. The reactivation of these genes in cancer samples makes them oncogene candidates, and their restricted expression profile in normal tissues makes them potential targets for therapy.

CT antigens were first identified by autologous typing^14^. The rapid development of PCR and microarray technology accelerated the identification processes based on the expression patterns of these genes^15^,^16^,^17^,^18^. To date, more than 200 genes have been recorded as *CT* genes in public databases^19^. Although great success has been achieved^17^,^18^,^19^, research on *CT* genes still faces many challenges. First, most studies have examined expression patterns in only a small number of samples^18^. The lack of transcriptomics data from a large number of normal tissue samples of multiple types as well as high-quality cancer samples has made it difficult to systematically and comprehensively evaluate these genes. Second, hundreds of *CT* genes are listed in databases; how they are reactivated and whether these reactivated testis proteins support tumorigenic features remains to be studied thoroughly. Some genes, such as *LIN28B*, have been reported to act as oncogenes in specific cancers^20^,^21^,^22^,^23^,^24^. However, the CT expression patterns of these genes have been ignored. Third, these genes have been pursued as targets for anticancer vaccines; however, researchers may have ignored the possibility that these genes could be useful targets of molecular-targeted therapy. For example, use of the CT antigen BRDT as a contraceptive target has been proposed due to its high efficacy and the minimal side effects associated with this therapeutic strategy^25^.

Recently, databases such as The Genotype-Tissue Expression (GTEx)^26^,^27^,^28^ and TCGA^29^ have provided a great deal of transcriptomic data from normal and tumour tissues and provided opportunities to identify *CT* genes and to explore the similarities and differences between spermatogenesis and tumorigenesis.

In this study, we performed a comprehensive, multiplatform analysis on large, independent and publically available databases (GTEx, HPM, TCGA, The Encyclopedia of DNA Elements (ENCODE), The Functional Annotation of The Mammalian Genome (FANTOM) and so on) and our data (NJMU-seq) to systematically identify and describe *CT* genes. We further defined extremely highly expressed *CT* genes (EECTGs) to indicate potential *epi-driver* genes and explore the correlation between these genes and mutations, promoter methylation levels and nearby non-coding RNAs (ncRNAs). After subsequent validation, we demonstrated that a meiosis-related EECTG (*MEIOB*) and its companion testis-specific ncRNA (TS-ncRNA; *LINC00254*) play crucial roles in carcinogenesis in lung adenocarcinoma (LUAD).

## Results

### Testis-specific genes and their enriched characteristics

**Figure 1:**
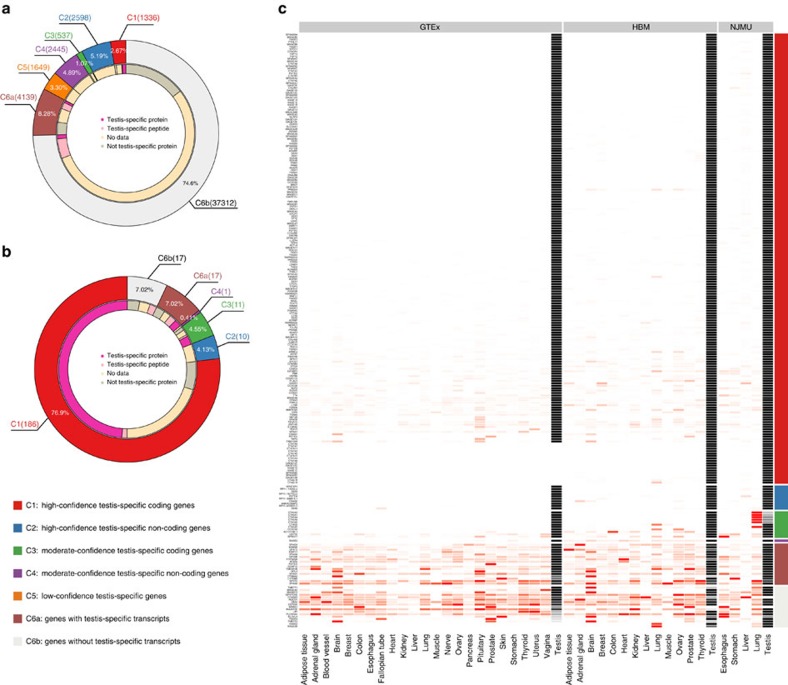
Classification of all genes and known *CT* genes. (**a**) All 50,016 genes were classified into six groups according to their mRNA expression patterns and classified into three groups according to their protein expression patterns. The pie chart displays the mRNA expression classification in the outer circle and the protein expression classification in the inner circle. (**b**) Pie chart summary of the classification of known *CT* genes. mRNA expression and protein abundance of known *CT* genes. The genes in the *y* axis were ranked by the SPM values from the GTEx database in descending order. The expression of each gene was rescaled to the interval [0, 1]. The depth of colour indicates the expression level. Black indicates testis and red indicates other normal tissues. The colour of each category corresponds with the colours in **a**,**b**. Genes were classified into the following six categories based on specificity measure (SPM) values: (C1) high-confidence testis-specific coding genes: GENCODE-annotated protein-coding genes (v19) with (**a**) SPM_GTEx_ >0.9, SPM_HBM_ >0.9 and SPM_NJMU_ >0.9; or (**b**) Known CT and SPM_GTEx_=0, SPM_HBM_ >0.9, SPM_NJMU_ >0.9 and gene copies with identical sequences; (C2) high-confidence testis-specific non-coding genes: GENCODE-annotated non-coding genes (v19) with SPM_GTEx_ >0.9, SPM_HBM_ >0.9 and SPM_NJMU_ >0.9; (C3) moderate-confidence testis-specific coding genes: GENCODE-annotated protein-coding genes (v19) with SPM_GTEx_ >0.9 and either SPM_HBM_ >0.9 or SPM_NJMU_ >0.9; (C4) moderate-confidence testis-specific non-coding genes: GENCODE-annotated non-coding genes (v19) with SPM_GTEx_>0.9 and either SPM_HBM_ >0.9 or SPM_NJMU_ >0.9; (C5) low-confidence testis-specific genes: genes with SPM_GTEx_ >0.9 but SPM_HBM_ ≤0.9 and SPM_NJMU_ ≤0.9; (C6) non-gene-level testis-specific gene: genes with SPM_GTEx_ ≤0.9. *G6* genes were then classified into the following two sub-groups using transcript abundance data from GTEx; (*C6a*) genes with testis-specific transcripts: C6 with SPM_GTEx transcript_>0.9; (*C6b*) genes without testis-specific transcripts: C6 with SPM_GTEx transcript_ ≤0.9. (**c**) Complete expression patterns of known CT genes in the GTEx, HBM and NJMU studies.

To identify *CT* genes at a genome-wide scale, we first defined testis-specific genes (TSGs) and classified all human genes into six categories based on expression patterns (Supplementary Fig. 1). We used three independent transcriptomic data sets from normal tissues (Supplementary Data 1) and defined TSGs including TS-ncRNAs. We found that 8,565 genes (17.12% of 50,016 GENCODE genes, Version 19) exhibited testis-specific expression patterns (Fig. 1a, Supplementary Data 2). Among them, 1,336 genes (2.67%) were protein-coding genes with higher confidence (C1) and were considered as candidate *CT* genes for further analysis. Among the genes not expressed predominantly in testis in the GTEx database (C6), some were classified as testis-specific transcripts (C6a, 8.28%); for these genes, at least one transcript was expressed exclusively in the testis. It is also worth noting that thousands of ncRNAs (10.08%, C2 and C4) showed testis-specific expression (Fig. 1a). Most of these non-coding genes (94.1%) were annotated as long ncRNAs (lncRNAs), pseudogenes or antisense RNAs based on the GENCODE v19 reference. Most of these genes (98.4%) did not share exons with protein-coding genes.

Using this classification, which included testis-specific transcripts, a majority (226, 92.98%) of the 243 known *CT* genes^19^ satisfied our criteria in the GTEx project (C1–C6a, Fig. 1b,c). However, seventeen known *CT* genes (C6b) were widely expressed in more than one tissue (Fig. 1b,c). Due to limitations in protein quantification technology, most previous studies defined TSGs at the mRNA level. The correlation between mRNA and protein expression is a matter of scientific debate, and studies have suggested that the process of translation from mRNA to protein is complicated. In this study, we used human proteomic data from 16 different normal adult tissues^30^ (Supplementary Data 1) to define testis-specific proteins (TSPs). Peptides mapped to multiple proteins may bias the evaluation of testis-specific expression. Some classical *CT* genes, such as genes from MAGE family, have similar sequences and shared peptides, making it challenging to define TSPs. To resolve this issue, we examined expression at both the protein and the peptide level in this analysis (Methods section). Of the 15,297 proteins annotated in the GENCODE databases, 1,218 proved to be TSPs; 418 were consistent with the TSGs from group C1 (Fig. 1a). The relatively small proteomic data sample size may have contributed to this inconsistency, especially for rarely expressed genes. Half of the TSGs in C1 were not expressed as protein in testis, indicating that there may be differences between mRNA estimations and protein abundance (for example, in quantification sensitivities or expression cutoffs). We cannot exclude the possibility that some TSGs may not be translated in the testis even though their mRNA level was high. We also observed TSPs without testis-specific mRNA expression. The occurrence of these genes may be due to the conservative alignment strategy of RNA-sequencing (RNA-Seq) data on genes with similar sequence (*CXorf49* and *CXorf49B*) or the presence of transcripts with different expression patterns (both testis-specific transcripts and non-testis-specific transcripts). Compared with the coding genes in C6b (non-TSGs), TSPs were significantly enriched as *C1* genes (enrichment ratio (ER)=23.47, Fisher's exact test *P*=6.01 × 10^−283^).

**Figure 2:**
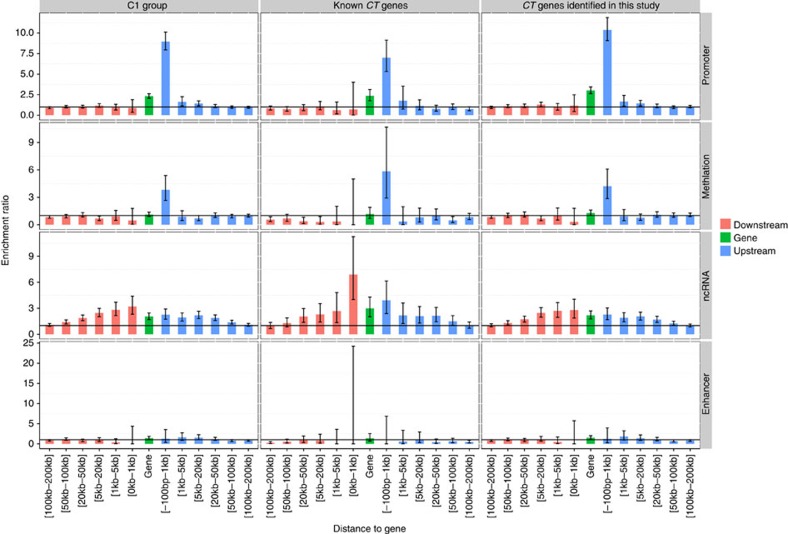
Enrichment analysis of testis-specific regulatory elements (TSREs). Enrichment analysis was used to evaluate the relationship between *TSREs* and *CT* genes. Four types of regulatory elements (promoters, methylation sites, non-coding RNAs and enhancers) were included in the analysis. The error bar represents the ER confidence interval.

We further examined whether regulatory elements (promoters, enhancers, ncRNAs or methylation sites) were involved in TSG expression patterns in the testis. By integrating data from Cap Analysis of Gene Expression analyses released by the FANTOM project and data from Reduced Representation Bisulfite Sequencing analyses released by the ENCODE project, we found that testis-specific promoters and demethylation sites were frequently located proximally upstream (−100 to 1 kb) of *C1* genes (ER_promoter_=10.10, Fisher's exact test *P*_promoter_=6.42 × 10^−253^; ER_methylation_=5.37, Fisher's exact test *P*_methylation_=6.57 × 10^−12^, Fig. 2). Furthermore, testis-specific ncRNAs were typically located 100 kb upstream or downstream of *C1* genes (Fig. 2). However, enrichment of testis-specific enhancers was not observed for *C1* genes (Fig. 2).

### *CT* genes and their reactivation in cancers

The TCGA project provides publically available expression profiles from thousands of cancer patients. Using these data, we distinguished *CT* genes from TSGs with higher confidence (*C1* genes). We included 19 cancer types in which expression abundance was observed from mRNA sequencing in our analysis (Supplementary Data 1). As listed in Supplementary Data 3, 1,019 (77%) of the 1,336 *C1* genes that were expressed in at least 1% of the samples (>5 normalized read counts) of any cancer type were *CT* genes for the particular cancer. We observed a similar enrichment pattern for regulatory elements for genes defined in our study as *C1* genes and known *CT* genes (Fig. 2). Previous studies have demonstrated that known *CT* genes are aberrantly activated in up to 40% of various cancer types^17^. Because of our large study sample size and advances in RNA sequencing technology^31^, we found that many *CT* genes (37.2–59.1%) were expressed in more than 40% of samples for each cancer type. For these genes, expression in most of the samples was relatively low; these genes may not play an important role in tumorigenesis. Because adjacent cancer tissues usually do not express *CT* genes, it was challenging to determine activated samples individually. Here we considered samples with extremely high expression (EE; Methods section) as samples activated for the *CT* gene. *CT* genes identified in at least 1% of EE samples were defined as EECTGs for a particular cancer. In total, 891 of the 1,019 identified *CT* genes passed these criteria for at least one cancer type (Supplementary Data 3); 300 EECTPs (extremely highly expressed CT proteins: EECTGs with TSP expression) were selected for further analysis.

**Figure 3:**
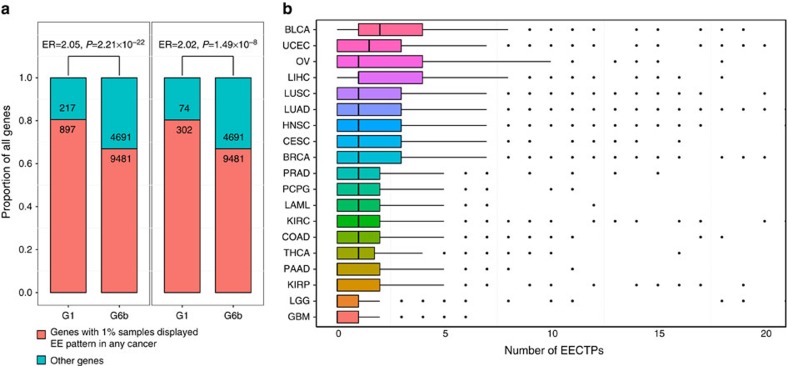
General description of EECTPs in 19 cancer types. (**a**) Enrichment analysis suggests that EE patterns were more likely to emerge in TSGs and TSPs, Fisher's exact test was applied to evaluate the enrichment ratio. (**b**) Number of activated EECTPs in 19 cancer types. The box plot displays the first and third quartiles (top and bottom of the boxes), the median (band inside the boxes), and the lowest and highest point within 1.5 times the interquartile range of the lower and higher quartile (whiskers).

We found that *C1* genes were approximately twice as likely to display this EE pattern in cancer samples compared with non-TSGs (ER=2.05, Fisher's exact test *P*=2.21 × 10^−22^, Fig. 3a). This finding indicates that TSGs were more likely to display this EE pattern in tumour tissues. Similar to the mutation spectrum observed in cancer^1^,^3^, the maximum EE frequency was <7% in each cancer type. Genes with EE patterns were very rare (median: 0–2) in each cancer sample. Some cancer types, such as pancreatic ductal adenocarcinoma, kidney renal papillary cell carcinoma, lower grade glioma and glioblastoma, presented with an average of 0 EECTPs and were considered to be ‘CT poor cancers' (Fig. 3b). We also measured *CT* gene expression in 24 LUAD samples by RNA sequencing and validated 19 EECTPs (7 novel, Supplementary Fig. 2).

For a more global analysis of TSGs and EECTPs, we performed a gene ontology analysis using the DAVID platform^32^. The top 10 clusters are shown in Supplementary Data 4. Genes involved in reproduction-related process were consistently enriched as TSGs and EECTPs. As the function of most *CT* genes in the development of cancer remained unknown, the information we obtained from the gene ontology term analysis was limited.

### Exclusive patterns of activation of EECTPs and mutations

**Figure 4:**
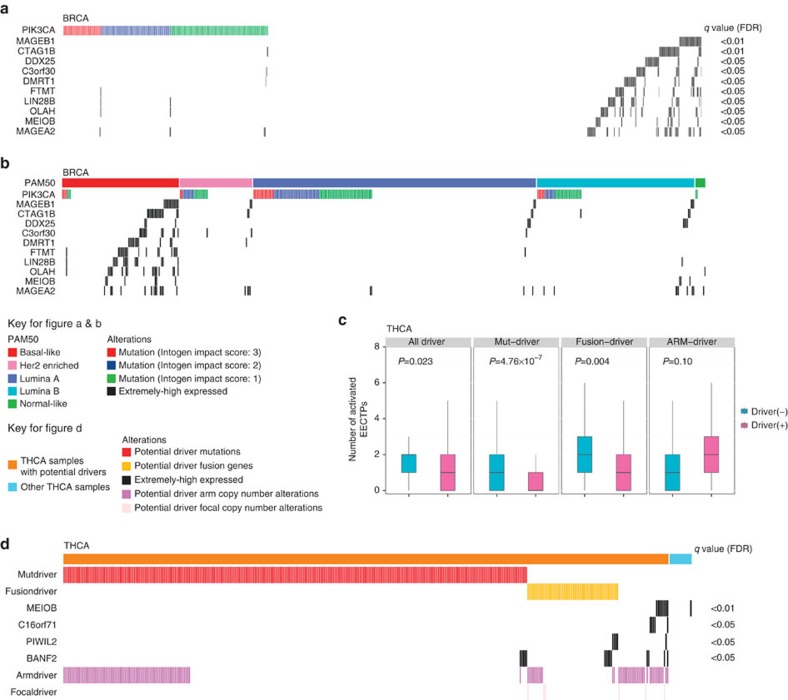
The association between the activation pattern of EECTPs and the alterations of the driver genes in patients with BRCA and papillary thyroid carcinoma. (**a**) Mutually exclusive pattern of EECTP activation and *PIK3CA* mutation in breast cancer. (**b**) Mutually exclusive pattern of EECTP activation and *PIK3CA* mutation in different PAM50 subtypes. (**c**) The total of number of activated EECTPs is significantly higher in papillary thyroid carcinoma patients without clear driver alterations (mutations and fusions). The box plot displays the first and third quartiles (top and bottom of the boxes), the median (band inside the boxes), and the lowest and highest point within 1.5 times the interquartile range of the lower and higher quartile (whiskers). (**d**) The activation of *MEIOB* is restricted in papillary thyroid carcinoma patients without driver mutations or fusions but co-occur with arm copy number drivers.

SMGs are commonly thought to be the major source of *mut-driver* genes^1^,^3^. Our aim in this study was to explore the relationship between the expression of EECTPs and the mutation of SMGs. Eight types of major cancers within the TCGA were selected for the analysis if more than 100 platform-overlapping samples were available. We used the proportion of somatic mutation numbers in SMGs and in all genes (the SMG mutation ratio) to represent the degree of samples driven by SMG mutation. We used the number of activated EECTPs to represent the proportion of samples driven by EECTPs. Interestingly, we found that the number of activated EECTPs was inversely associated with the SMG mutation ratio after adjusting for cancer type (*β*=−4.58, linear regression *P*=8.28 × 10^−5^, Supplementary Fig. 3a). However, no significant association between the number of activated EECTPs and the mutation ratio of EECTPs was observed (Supplementary Fig. 3b). In many cancers, we noticed that the number of activated EECTPs was significantly higher in *TP53* mutated samples (adjusted by false discovery rate (FDR), *P*<0.05, Supplementary Fig. 4a). Because *TP53* is a gatekeeper for genome instability, we excluded *TP53* gene mutations when calculating the SMG mutation ratio, which resulted in a more obvious negative correlation (*β*=−5.75, linear regression *P*=6.87 × 10^−7^, Supplementary Fig. 3c). We further evaluated the correlation between the mutation pattern of each SMG and the activation of each EECTP. Because the frequency of mutations and EECTPs' activation were both low, we performed this analysis only in breast cancer (BRCA), which had the largest sample size. Eight SMGs were significantly associated with the activation of EECTPs (Supplementary Fig. 4b). However, only *PIK3CA* was consistently associated with the activation of EECTPs in multiple molecular subtypes (Supplementary Fig. 4c). As *PIK3CA* is a well-known BRCA oncogene, we further explored the correlation between each EECTP and mutations in *PIK3CA* (Supplementary Data 5). This analysis revealed that the relationship between the activation of multiple EECTPs was mutually exclusive with mutations in *PIK3CA* (FDR adjusted, *P*<0.05, Fig. 4a); these findings included *MEIOB*. *CT* gene expression was enriched in basal-like BRCA samples, which have a lower *PIK3CA* mutation frequency (Fig. 4b). This suggests that the activation of EECTPs may also be related to molecular subtypes of BRCA. A recent study identified multiple *mut-driver* genes in a handful of samples and clearly defined mutually exclusive driver genes for patients with papillary thyroid carcinoma. This provided us with an opportunity to study the correlation between EECTPs and *mut-driver* genes. As expected, the total of number of activated EECTPs was significantly higher in patients without clear driver alterations (Fig. 4c). Moreover, we identified four EECTPs prone to activation in patients without driver mutations. We observed that the activation of *MEIOB* was restricted in patients without driver mutations or fusions. Almost all *MEIOB*-activated patients had driving arm-level copy number alterations. This evidence suggests that the activation of EECTPs may complement alterations in known driver genes.

### EECTPs may be regulated by promoter methylation levels

**Figure 5:**
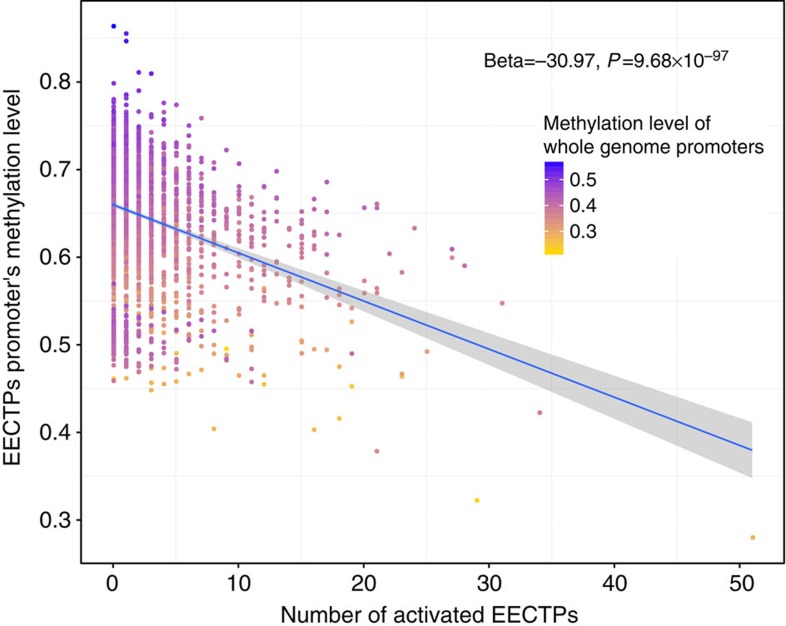
Negative correlation between the average promoter methylation level of EECTPs and the number of activated EECTPs. The depth of colour indicates the genome-wide promoter methylation level. The blue line is the best-fit linear regression and the shaded area indicates the confidence interval.

By integrating DNA methylation data from TCGA, we further explored the relationship between the activation of EECTPs and the methylation level of their promoters. Seven cancer types were selected from the major TCGA cancers with more than 100 platform-overlapped samples and were included in our analysis. The average promoter methylation levels of EECTPs were negatively associated with activated EECTP numbers (*β*=−30.97, linear regression *P*=9.68 × 10^−97^, Fig. 5) after adjusting for cancer type. When the average promoter methylation level of all genes was considered to exclude the influence of genome-wide hypomethylation, the negative association remained (*β*=−56.38, linear regression *P*=1.68 × 10^−52^).

We further compared methylation levels between EE and non-EE samples for each EECTP in the corresponding cancer type. For LUAD, BRCA and head and neck squamous cell carcinoma, more than half of EECTPs exhibited differential methylation levels between EE and non-EE samples (Supplementary Data 6). We validated seven EECTPs in our LUAD samples. Of these, two genes (*TSPY1* and *RBMY1A1*) were located on the Y chromosome; promoter methylation information was not included in the Illumina 450 k methylation array. Two genes proved to be significantly demethylated in EE samples (*RHOXF1* and *VCX3B*, Supplementary Fig. 5), suggesting that these genes may be reactivated through aberrant promoter methylation. The remaining genes (*LIN28B*, *MEIOB* and *SPATA22*) may be activated by an alternative mechanism in LUAD.

### CT ncRNA

As noted above, we found thousands of non-coding genes with testis-specific expression (TS-ncRNAs; C2 and C4). To better understand the expression patterns of TS-ncRNAs in cancer samples, we used two data sets from recently published papers. One study provided us with expression abundance data for 1,791 differentially expressed ncRNAs in TCGA^33^. We found that 239 of these ncRNAs were TS-ncRNAs (namely, CT-ncRNAs; Supplementary Data 7). Another study performed *de novo* assembly of ncRNAs from the TCGA data and released expression abundance data for cancer/lineage-associated ncRNAs^34^. Based on these differentially expressed ncRNAs, we successfully annotated 967 ncRNAs using transcripts from GENCODE v19; annotations were performed using coordinates that overlapped with the reference ncRNAs (Supplementary Methods). Of these ncRNAs, 173 were CT-ncRNAs (Supplementary Data 7). We further explored whether the expression of CT-coding genes was influenced by CT-ncRNAs and found 174 and 258 cancer-specific CT-coding gene/CT-ncRNA pairs (where the distance between the CT-coding gene and CT-ncRNA was <100 kb) from the two studies. Many pairs displayed significant correlation (*P*_FDR_<0.05), suggesting that close relationships exist between CT-coding genes and CT-ncRNAs (Supplementary Data 8). Among these CT-coding gene/CT-ncRNA pairs, some genes have been reported to participate in cancer or spermatogenesis. For example, *DEPDC1* was reported to contribute to bladder cancer oncogenesis by interfering with the transcriptional repressor *ZNF224* (ref. 35). *PWRN1* was reported to be a part of the IC-SNURF-SNRPN transcription unit, which may be involved in the process of spermatogenesis^36^. Without the expression data for all of ncRNA transcripts in the TCGA, we cannot evaluate focally activated ncRNAs comprehensively. Using our LUAD samples, we identified a TS-ncRNA (*LINC00577*) that was correlated with the expression of *LIN28B* (Spearman's rank correlation coefficient=0.42, *P*=0.04). This finding suggests that *CT* genes may be activated by nearby TS-ncRNAs.

### *MEIOB* is a meiosis-related epi-driver candidate in LUAD

**Figure 6:**
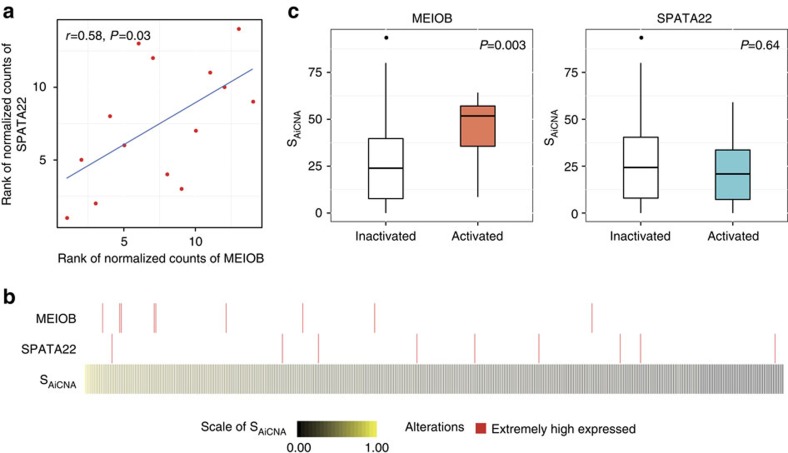
*MEIOB* and *SPATA22* co-expression patterns in testis and tumour samples and the association between MEIOB activation and S_Ai_. (**a**) *MEIOB* and *SPATA22* are co-expressed in testis. (**b**) *MEIOB* and *SPATA22* are exclusively activated in LUAD samples. (**c**) Activation of *MEIOB* is associated with higher S_Ai_. The box plot displays the first and third quartiles (top and bottom of the boxes), the median (band inside the boxes), and the lowest and highest point within 1.5 times the interquartile range of the lower and higher quartile (whiskers).

Of the seven EECTPs mentioned above, we observed that *MEIOB* and *SPATA22* were recently reported to be essential factors acting during meiotic recombination. MEIOB exhibits 3′–5′ exonuclease activity and localizes to meiotic chromatin by forming a complex with SPATA22. Thus, MEIOB is essential for meiotic recombination. SPATA22 may modulate the nuclease activity and substrate specificity of MEIOB^37^. First, we explored the co-expression patterns of these two genes in testis and tumour tissues. We found that the expression of *MEIOB* and *SPATA22* was significantly correlated in 14 GTEx normal testis tissues (Fig. 6a). Most interestingly, the EE patterns of these two genes were mutually exclusive in patients with LUAD (Fig. 6b). We also observed similar *MEIOB* and *SPATA22* activation in other tumour types (Supplementary Fig. 6).

In addition, the expression of *MEIOB* was positively correlated with the genome-wide burden of focal copy number aberrations (CNAs) among samples from 10 cancer types with *MEIOB* and *SPATA22* activation (Spearman's rank correlation coefficient=0.12, *P*=8.30 × 10^−14^). The correlation was significant (*P*<0.05) in LUAD samples and samples from other cancer types (bladder urothelial carcinoma: *P*=0.008; BRCA: *P*=0.02; thyroid carcinoma: *P*=0.05; uterine corpus endometrial carcinoma: *P*=0.01). To unravel the potential aneuploidy formation mechanism in which *MEIOB* may be involved, we recalled allele-specific copy number alternations and calculated the score of allele-imbalanced events using the method described previously^38^. Similar to a previous report concerning *HORMAD1* (ref. 38), we found that the activation of *MEIOB* significantly increased the signal of allele-imbalanced alternations (the score of allele-imbalanced CNAs and copy-neutral loss of heterozygosity (CnLOH)) in LUAD samples (Fig. 6c).

**Figure 7:**
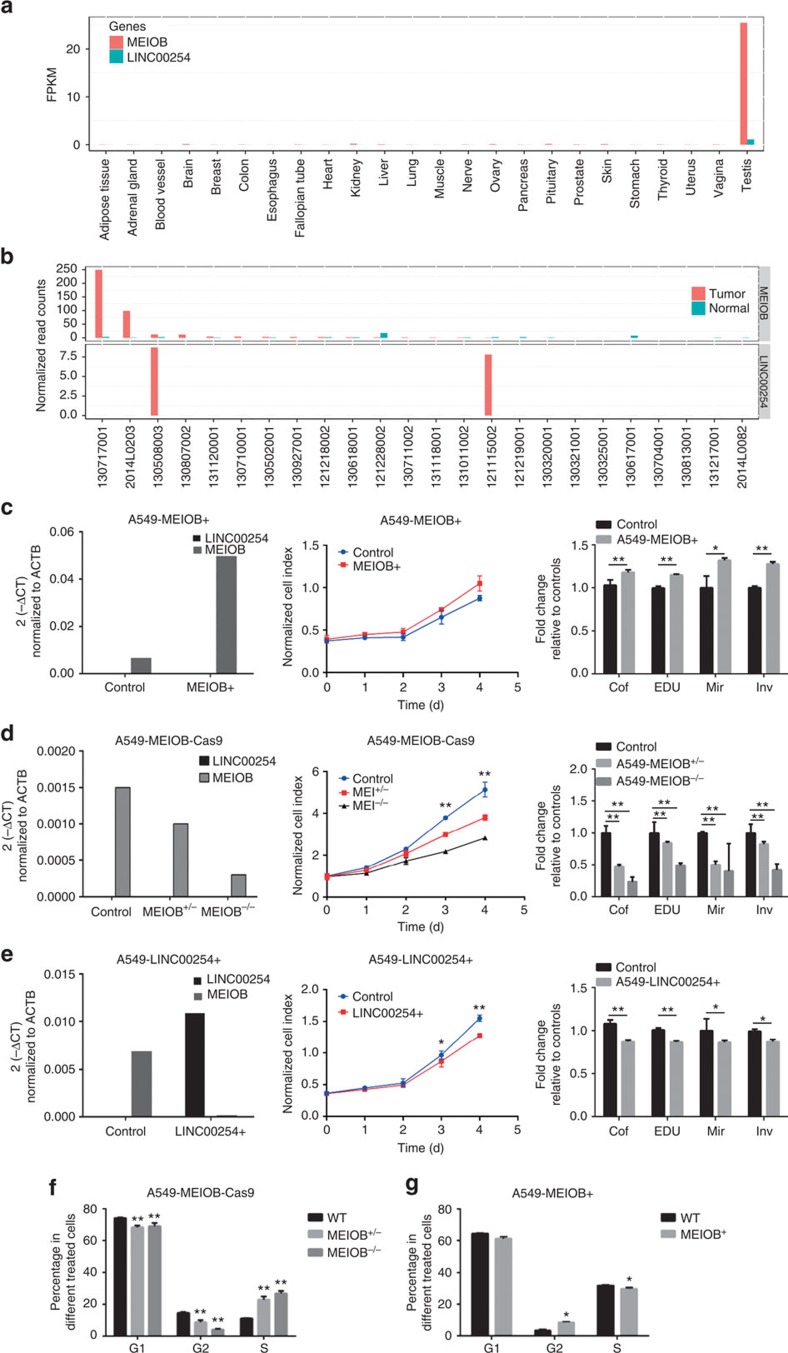
*MEIOB/LINC00254* drive lung cancer cell growth, migration and invasion *in vitro*. (**a**) Expression of *MEIOB*/*LINC00254* in the GTEx data sets. (**b**) Expression of *MEIOB*/*LINC00254* in our 24 tumour/normal paired samples. (**c**) Overexpression of *MEIOB* effects A549 vitality. (**d**) Knockout of *MEIOB* effects A549 vitality. (**e**) Overexpression of *LINC00254* effects A549 vitality. Relative expression of *LINC00254* and *MEIOB* (left) and a growth curve (middle) of different treated A549 cells. The fold change is relative to the control of colony formation; EdU staining, migration and invasion are shown together (right). (**f**) Cell cycle distribution in the A549 control and *MEIOB* knockout cells. (**g**) Cell cycle distribution in the A549 control and *MEIOB* overexpressing cells. Error bars represent s.e.m, *n*=5. *P<0.05 compared with the vector control. **P<0.001 compared with the vector control. All of the experiments were repeated three times.

To prove that MEIOB plays a critical role during the development of cancer, we designed a series of *in vitro* experiments. Expression pattern of *MEIOB* in normal and LUAD tissues were displayed in Fig. 7a,b. The overexpression of *MEIOB* in A549 cells resulted in increased cell viability and malignant phenotypes. Deletion of *MEIOB* inhibited clonogenicity as well as cell growth, invasion and migration (Fig. 7c,d, Supplementary Fig. 7). We further demonstrated that the overexpression of *MEIOB* in A549 cell lines resulted in a significantly reduced population of S phase cells and an increased population of G2 phase cells. Deletion of *MEIOB* led to opposite results. In addition to coding genes, we identified a TS-ncRNA (*LINC00254*) near *MEIOB*. *LINC00254* is located 6 kb upstream of *MEIOB*. It is a testis-specific expressed transcript (Fig. 7a) and is expressed in samples without *MEIOB* activation (Fig. 7b); however, *LINC00254* was not expressed in any of the LUAD cell lines tested (Supplementary Fig. 8a,b). In the A549 cell line, the stable overexpression of *LINC00254* downregulated *MEIOB* and produced results similar to those seen in the *MEIOB* knockout (Fig. 7e, Supplementary Figs 7 and 8c). We further performed cell cycle assay in *MEIOB* overexpressing and knockout A549 cell lines. We observed that the overexpression of *MEIOB* in A549 cell lines resulted in significantly reduced population of S phase cells and increased population of G2 phase cells (Fig. 7f). On the contrary, the deletion of *MEIOB* led to opposite results (Fig. 7g). These results suggest that *MEIOB* may participate in the process of tumorigenesis by damaging genome stability. *LINC00254* may act as a natural inhibitor of *MEIOB*.

## Discussion

It has long been acknowledged that the processes of germ cell development and tumour development share important similarities, including immortalization, the induction of meiosis, invasion and migration^12^. The products of these gamete-specific genes may be deleterious for the orderly requirements of normal somatic cells and highly advantageous in cancer cells. Thus, the systematic evaluation of the transcriptomes of germ cells and tumour samples may help us identify key elements involved in both gametogenesis and tumorigenesis and understand their differences. In the present study, we integrated multiple independent transcriptome databases containing both normal and tumour samples and systematically explored the molecular landscape of *CT* genes. Detailed information concerning the *CT* genes mentioned in this article is freely accessible through our search engine (CTatlas: http://reprod.njmu.edu.cn/ctatlas/).

*CT* genes were first observed and considered to be candidate targets for immunotherapy due to their immunogenicity. Since then, immunogenicity has been regarded as one of the most important characteristics of *CT* genes. However, 18 of the 23 phase II/III clinical trials that tested 17 distinct therapeutic anticancer vaccines failed to achieve their primary objectives; this included a trial for the vaccine targeting MAGEA3 in non-small cell lung cancer^39^,^40^,^41^. Frustrations concerning clinical experiments indicate the great challenges present in the development of immunotherapy without the sufficient identification of the expression patterns of *CT* genes. Further characterization of the role of epi-drivers and the reactivation mechanism of *CT* genes would greatly benefit the development of therapeutic strategies.

With the development of cancer genomics, molecular-targeted therapies are increasingly used as an alternative to chemotherapy^42^,^43^. The development of targeted therapies requires the identification of targets that play a key role in cancer cell growth and survival. Because *CT* genes are normally expressed exclusively in the testis but frequently turned on in many types of tumours, there is a significant possibility that *CT* genes are involved in the process of tumorigenesis. *CT* genes are therefore considered to be epi-driver candidates, which have been described as one of the major sources of ‘dark matter' in cancer. MAGEA3 serves as an example; although a clinical trial of an immunotherapy targeting MAGEA3 was suspended because it failed to improve the survival of patients with non-small cell lung cancer, MAGEA3 was recently reported to be necessary for cancer cell viability and sufficient to drive the tumorigenic properties of non-cancerous cells^44^. Therefore, efforts to inhibit MAGEA3 should not be halted. Rather, failed approaches should be replaced with a more suitable individualized therapy plan. In our study, we considered genes that were extremely highly expressed in more than 1% of the samples as potential candidates for epi-drivers. Consistent with previous studies^44^, MAGEA3 was defined as an EECTP in multiple cancer types (BRCA, colon adenocarcinoma, glioblastoma and so on, Supplementary Data 3). Thus, cases with extremely high *MAGEA3* expression in our study may be ideal candidates for a future therapy targeting MAGEA3 and may benefit from such treatment.

Meiosis in gametogenesis and aneuploidy in tumorigenesis share similar characteristics and mechanisms^12^. The induction of DNA double-strand breaks, which increases genome diversity and is essential for proper chromosome segregation at the first meiotic division^45^, is an important process during meiotic recombination. Mitosis inhibits this process; mitosis can lead to telomere fusions that produce dicentric chromosomes and aneuploidy, especially in the presence of exogenous genotoxic stress^46^. A recent study identified a *CT* gene involved in the promotion of nonconservative recombination in meiosis as a novel driver of an allelic imbalance phenotype in triple-negative BRCA^38^. In this study, we also observed the activation of a *CT* gene, *MEIOB*. MEIOB exhibits 3′–5′ exonuclease activity during meiotic recombination and is essential for meiosis^37^. This activity may contribute to the genome instability observed in LUAD patients. The co-factor for *MEIOB* (*SPATA22*) in meiosis recombination, which was co-expressed with *MEIOB* in testis, displayed mutually exclusive expression pattern in samples with LUAD and other cancer types. This reflects the common and unique characteristics of germ and tumour cells. In addition, we found that ncRNAs with testis-specific expression patterns could possibly contribute to carcinogenesis by interacting with these *CT* genes. These ncRNAs can either promote (*LIN28B* and *LINC00577*) or inhibit (*MEIOB* and *LINC00254*) nearby genes.

We also found that EECTPs may be exclusively activated by mutations in SMGs. In BRCA, this mutually exclusive pattern between *PIK3CA* and EECTPs achieved statistical significance due to the sufficient sample size. In all cancer types, we found some samples without mutations in SMGs^1^; therefore, *CT* genes may serve as complements of mut-drivers. This hypothesis was further strengthened by data from a genomic study on papillary thyroid carcinoma. Our results revealed that demethylation was an important mechanism for the reactivation of *CT* genes, which was consistent with conventional knowledge of *CT* gene activation mechanisms^13^,^47^.

In conclusion, our study identified hundreds of *CT* genes in 19 cancer types using publically available databases. We successfully extended the definition of CT antigens to include *CT* genes (CT proteins and CT-ncRNAs). We used EE patterns to define and identify EECTGs and successfully proved that EECTGs, which might be activated by promoter demethylation or by proximal CT-lncRNAs, could serve as potential sources of epi-drivers in cancer. Generally speaking, these findings expand our knowledge of *CT* genes and provide new perspectives for identifying *epi-driver* genes in cancer.

## Methods

### Public data sets used in this study

We used the multiple public databases involving both normal and tumour tissues to evaluate TSGs and evaluate cancer-specific *CT* genes and EECTGs. Detailed information of these databases was listed in the Supplementary Table 1 and Supplementary Data 1.

### Classification of all genes

A total of 50,016 unique genes with expression abundance data (FPKM) from three data sets (GTEx, Illumina Human Bodymap and NJMU-seq) were included in the analysis after integration. ENSEMBL ID (GENCODE v19) was used as an indicator. Genes were classified into the following six categories (Fig. 1) based on specificity measure (SPM) values:

(C1) high-confidence testis-specific coding genes:

GENCODE-annotated protein-coding genes (v19) with

- SPM_GTEx_ >0.9, SPM_HBM_ >0.9 and SPM_NJMU_ >0.9; or
- Known CT and SPM_GTEx_=0, SPM_HBM_ >0.9, SPM_NJMU_ >0.9 and gene copies with identical sequences;

(C2) high-confidence testis-specific non-coding genes: GENCODE-annotated non-coding genes (v19) with SPM_GTEx_ >0.9, SPM_HBM_ >0.9 and SPM_NJMU_ >0.9;

(C3) moderate-confidence testis-specific coding genes: GENCODE-annotated protein-coding genes (v19) with SPM_GTEx_ >0.9 and either SPM_HBM_ >0.9 or SPM_NJMU_ >0.9;

(C4) moderate-confidence testis-specific non-coding genes: GENCODE-annotated non-coding genes (v19) with SPM_GTEx_ >0.9 and either SPM_HBM_ >0.9 or SPM_NJMU_ >0.9;

(C5) low-confidence TSGs: genes with SPM_GTEx_ >0.9 but SPM_HBM_ ≤0.9 and SPM_NJMU_ ≤0.9;

(C6) non-gene-level TSG: genes with SPM_GTEx_≤0.9.

To further distinguish genes with testis-specific expression patterns at the transcript level but not at the gene level, we classified G6 into the following two sub-groups using transcript abundance data from GTEx; (C6a) genes with testis-specific transcripts: C6 with SPM_GTEx transcript_ >0.9; (C6b) genes without testis-specific transcripts: C6 with SPM_GTEx transcript_ ≤0.9. Details of SPM value calculations are provided in the Supplementary Information (Methods to evaluate TSGs).

To accurately define TSPs, we used expression data curated from gene-specific peptides. We used same SPM criteria to define TSPs (Supplementary Figure 9). Some classical *CT* genes had similar sequence and shared peptides, such as MAGE family genes, would be missed. To address this issue properly, we used expression profiles at both the protein level and the peptide level and classified all genes into the following four groups:

1. TSPs
2. Testis-specific peptides
3. Proteins that had at least one peptide with testis-specific expression (SPM >0.9).
4. No data: proteins or genes with no protein expression data.
5. Non-TSPs: other proteins.

### Evaluation of TSGs/*CT* genes related to testis-specific regulatory elements

Enrichment analysis was applied to evaluate the relationship between TSGs/*CT* genes and their nearby testis-specific regulatory elements (TSREs). Four types of TSREs (promoters, methylation levels, ncRNAs and enhancers) were considered. Detailed information concerning the definition and identification of TSREs is listed in the Supplementary Methods. Fisher's exact test was applied to evaluate the ER, which was calculated as follows:


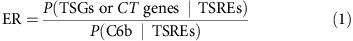


### Criteria for cancer-specific *CT* genes and EECTGs/EECTPs

Genes were further grouped as *CT* genes and EECTGs/EECTPs after considering the above six categories and cancer-related information. *CT* genes met the following criteria: (1) exhibited testis-specific expression patterns with high confidence (C1); (2) exhibited expression (>5 normalized read counts) in at least 1% of cancer samples. EECTGs met the following criteria: (1) exhibited *CT* gene expression patterns; (2) exhibited EE (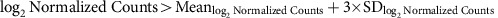) in at least 1% of cancer samples. EECTPs met the following criteria: EECTGs exhibiting testis-specific expression patterns at the protein level.

Go analysis was performed using DAVID online tools ( http://david.abcc.ncifcrf.gov/). The top 10 enriched clusters were determined by automated functional annotation clustering with default parameters.

### Evaluation of the relationship between mutations and EECTPs

As driver mutations are significantly less frequent than passenger mutations, we used the total mutation number to reflect the burden of passenger mutations. We calculated mutation ratio as follows:


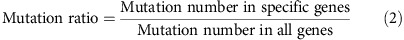


The mutation ratio represents the degree of samples driven by mutations for specific genes. The activated number of EECTPs represents the degree of samples driven by EECTPs. Linear regression was used to evaluate the association between the mutation ratio and the activated number of EECTPs. For each SMG, a Wilcoxon's rank-sum test was used to compare the activated EECTP numbers between mutated and non-mutated samples. Fisher's exact test was employed to test mutually exclusive patterns between the mutation patterns of SMGs and EE patterns of EECTPs.

### Relationship between promoter methylation levels and EECTPs

The default RnBeads^48^ workflow was used for quality control and to preprocess raw idat files produced by the Infinium HumanMethylation450 BeadChip Kit. Beta values for the promoters were extracted from the RnBeads output. Mean beta values were calculated to reflect promoter methylation levels for all EECTPs or for all genes. Linear regression models were applied to evaluate the relationship between promoter methylation and the activated number of EECTPs. For each EECTP, a Wilcoxon's rank-sum test was used to compare the promoter methylation levels between activated and inactivated samples.

### DNA copy number analysis and chromosomal instability scarring scoring

The global burden of focal CNAs was calculated as the total length of focal copy number (gain and loss) for each individual. The cutoffs for parameter Seg.CN were set to −0.2 and 0.2 to distinguish loss and gain, respectively. Allele-specific copy number analysis was performed with allele-specific copy number analysis of tumours (ASCAT v2.1) (ref. 49). Segments of allele-specific copy number profiles were classified into one of the following three non-overlapping types: allele-imbalanced CNAs (AiCNAs), allele-balanced CNAs (AbCNAs) or CnLOH. We calculated the score of each category (S_AiCNA_, S_AbCNA_ and S_CnLOH_) according to methods described previously^38^. S_Ai_ was calculated by summing S_AiCNA_ and S_CnLOH_, thereby capturing all allelic imbalance events. A Wilcoxon's rank-sum test was used to compare the global burden of focal CNAs, or S_Ai_, between the activated and the inactivated samples.

### In-house mRNA expression data sets

We obtained normal human tissue samples (*n*=7, 5 normal tissues, Supplementary Data 1) to define TSGs. We paired these samples with tumour/adjacent tumour tissues from LUAD patients (*n*=24) to validate EECTGs expression patterns. Human tissue samples used for mRNA expression analyses in the NJMU study were collected and handled in accordance with Chinese laws and regulations and were obtained from Affiliated Hospitals of Nanjing Medical University. At recruitment, informed consent was obtained from each subject. Tissues samples were preserved using RNA-later solution. Haematoxylin and eosin-stained sections from each sample were examined by a board-certified pathologist to ensure sampling of representative normal tissue. Total RNA was extracted from tissue samples using the RNeasy Mini Kit (Qiagen, Hilden, Germany) according to the manufacturer's instructions. The extracted RNA samples were analysed using an Agilent 2100 Bioanalyzer system (Agilent Biotechnologies, Palo Alto, USA) with the RNA 6000 Nano Labchip Kit. Only samples of high-quality RNA (RNA integrity number ⩾7.5) were used in the subsequent mRNA sample preparation for sequencing. PolyA-minus RNAs were fractionated from total RNA samples and RNA-Seq libraries were generated by RNA-fragmentation, random hexamer-primed complementary DNA (cDNA) synthesis, linker ligation and PCR amplification using a TruSeq RNA Sample Prep Kit (Illumina, Inc.). The purified DNA libraries were sequenced with an Illumina HiSeq1500 platform (paired-end, 100 base). To maintain consistency with the estimated expression abundance method used for the GTEx and TCGA databases, we estimated the FPKM for normal tissues to evaluate TSGs using the TopHat-Cufflinks protocol (TopHat v2.0.9 and Cufflinks v2.2.1) and normalized read counts (normalized by upper-quantile method)^50^ for paired tumour/adjacent tumour tissues to validate EE patterns using the RSEM (RSEM v1.2.12). GENCODE v19 was used as the reference genome. The RNA-Seq data have been deposited at ArrayExpress (E-MTAB-4063).

### Cell culture

Human lung cancer cell line A549 was cultured in RPMI 1640 (Gibco) supplemented with 10% FBS. Cells were maintained at 37 °C with 5% CO_2_. The sources of the cell line and mycoplasma contamination were evaluated by Beijing JianLian Gene Technology Co., Ltd, on September 2013. DNA prepared from our A549 cells using a commercial Chelex100 kit was analysed by STR (Short tandem repeat) profiling. Our A549 cell line is considered to be ‘identical' to a culture in the ATCC A549 STR database; the entered STR profile yields a 100% match (11 of 11 loci) to the result set. No cross-contaminated cell lines or mycoplasma contamination was detected. Lentiviral infection was performed as follows: HEK-293T cells were co-transfected with pHelper 1.0 and pHelper 2.0 constructs and packaging plasmids. The progeny viruses released from HEK-293T cells were filtered, collected with a centrifugal filter unit (Centricon Plus-20, P31925, Millipore) and used to infect A549 cells. The lentivirus infected these two cancer cells via 8 mg ml^−1^ Polybrene mixed in RPMI 1640.

### RNA isolation and quantitative real time-PCR

Total RNA was extracted using TRIzol Reagent (Invitrogen) and reverse transcribed using the High Capacity RNA-to-cDNA Kit (Takara). cDNA was quantified using TaqMan Gene Expression Master Mix through an ABI 7900HT System (Applied Biosystems; Expression TaqMan lot number: *MEIOB*-1119967; *LINC00254*-1175109).

### Protein isolation and western blotting

Cells were lysed in mammalian protein extraction reagent (Beyotime). After quantification using a BCA protein assay kit (Beyotime), total proteins were separated by SDS–PAGE under denaturing conditions and transferred to PVDF membranes (Millipore). Membranes were blocked in 5% non-fat milk and incubated with and anti-MEIOB (ab150886, respectively; Abcam), followed by incubation with anti-rabbit secondary antibodies. We used seeBlue@ Plus 2 Prestained (lot#:1368753) as indicators of proteins. Immunoreactive proteins were visualized using a molecular imager (Bio-Rad). Uncropped scans of western blots were included in the Supplementary Fig. 8d.

### Gene deletion

Guide RNAs were designed to recognize chr16:1911998–1912070 (*MEIOB*) and chr16:1928364–1934279 (*LINC00254*) (hg19) and cloned into PGL3. Constructs were introduced into lung cancer cell lines (A549) using Lipofectamine 2000 reagent (Invitrogen) along with a plasmid encoding Cas9. Cells were sorted using a flow cytometer to capture cells with high green fluorescent protein signals. Colonies were grown from single cells. Complete deletion of both lncRNA alleles was confirmed by PCR using primers flanking lncRNA. The deletion of these two genes was confirmed by both sequencing and western blotting. Single guide RNA (sgRNA) sequences and primers used for amplifying the sgRNA target site and for sequencing are listed in Supplementary Table 2.

### Cell proliferation assay

Treated cells were seeded in 96-well plate and the viability of cells was measured with a Cell Counting Kit-8 (CCK8, Dojindo, Japan) daily for 5 days. Briefly, 10 μl of CCK8 solution was added to each well with 100 μl RPMI 1640 and incubated at 37 °C for 2 h. The absorbance was measured at wavelengths of 450 nm. We also used an 5-ethynyl-2'-deoxyuridine (EdU) array to confirm the cell viability between differently treated cells. According to the manual of a EdU labelling/detection kit (Ribobio, Guangzhou, China), 50 M EdU labelling medium was added to the cell culture to allow incubation for 2 h at 37 °C under 5% CO_2_. Subsequently, cultured cells were fixed with 4% paraformaldehyde (pH 7.4) for 30 min and incubated with glycine for 5 min. After washing with PBS, staining with anti-EdU working solution was performed at room temperature for 30 min. Following washing with 0.5% TritonX-100 in PBS, the cells were incubated with 5 g ml^−1^ Hoechst 33342 dye at room temperature for 30 min, followed by observation under a confocal laser scanning microscope (ECLIPSE-Ti, Nikon). The percentage of EdU-positive cells was calculated from nine random fields in three wells. The results are given as mean±s.d. of cell number relative to the vehicle control.

### Colony formation assay

Cells were cultured in six-well plates (300 cells per well) for 3 weeks and then fixed with methanol for 20 min at room temperature. After fixation, colonies were stained with 0.5% crystal violet for 30 min. Images of all wells were captured at room temperature and were counted by hand with the aid of imaging software. The results are given as mean±s.d. of cell colonies relative to the vehicle control.

### Migration and invasion

Cell migration and invasion were studied using Costar Transwell plates (6.5 mm diameter insert, 8.0 mm pore size, polycarbonate membrane; Corning Sparks, MD). Lung cancer cells were plated at a density of 2 × 10^4^ cells per 200 μl in the Transwells 1 day for migration and 2 days for invasion before the migration experiment. The cells were then fixed with methanol for 20 min at room temperature. After fixation, colonies were stained with 0.5% crystal violet for 30 min. The membranes were then dried, inverted and mounted on microscope slides for analysis. Images of four random fields for each membrane were captured at room temperature via a Nikon microscope and were counted by hand with the aid of imaging software. Counts from all 10 fields were averaged to obtain a mean cell count for each membrane. All experiments were performed at least three times with *n*=3 per trial. The results are given as mean±s.d. of cell migration and invasion relative to the vehicle control.

### Cell cycle

The cell cycle was analysed by flow cytometry. For cell cycle analysis, transfected lung cancer cells were suspended in 75% ethanol overnight and centrifuged at 1,000 r.p.m. Following two washes of the cell pellets in PBS, 50 mg ml^−1^ propidium iodide (PI) and 100 g ml^−1^ DNase-free RNase A was added and the cell pellet was re-suspended. Following incubation for 30 min at 37 °C, the samples were analysed by flow cytometry. Cell cycle distribution was further analysed with Cell Quest software (Becton Dickinson, San Jose, CA) and Mod Fit LT (Verity Software House, Topsham, ME).

## Additional information

**Accession codes:** The RNA-Seq data have been deposited in the ArrayExpress database under accession code E-MTAB-4063.

**How to cite this article:** Wang, C. *et al*. Systematic identification of genes with a cancer-testis expression pattern in 19 cancer types. *Nat. Commun.* 7:10499 doi: 10.1038/ncomms10499 (2016).

## Supplementary Material

Supplementary Information Supplementary Figures 1-9, Supplementary Tables 1-2, Supplementary Methods and Supplementary References.

Supplementary Data 1 Information on the samples used in this study.

Supplementary Data 2 Proteins without unqiue peptides but all peptides were testis-specific expressed.

Supplementary Data 3 Information of CT genes and EECTGs defined in our study: For each CT gene defined in our study, the percentage of expressed samples (normalized read counts>5) and mean±SD of log2-transformed expression abundance are listed.

Supplementary Data 4 Functional annotation clustering of TSGs (G1).

Supplementary Data 5 Mutations on PIK3CA in BRCA samples.

Supplementary Data 6 EECTPs with significantly different promoter methylation level between activated and inactivated samples.

Supplementary Data 7 List of CT-ncRNAs defined using data from the lncrnator.

Supplementary Data 8 Correlation between CT coding genes and CT-ncRNAs (lncrnator).

## References

[b1] Kandoth C. . Mutational landscape and significance across 12 major cancer types. Nature 502, 333–339 (2013). 24132290 10.1038/nature12634 PMC3927368

[b2] Lawrence M. S. . Discovery and saturation analysis of cancer genes across 21 tumour types. Nature 505, 495–501 (2014). 24390350 10.1038/nature12912 PMC4048962

[b3] Vogelstein B. . Cancer genome landscapes. Science 339, 1546–1558 (2013). 23539594 10.1126/science.1235122 PMC3749880

[b4] Pe'er D. & Hacohen N. Principles and strategies for developing network models in cancer. Cell 144, 864–873 (2011). 21414479 10.1016/j.cell.2011.03.001 PMC3082135

[b5] Stratton M. R. Journeys into the genome of cancer cells. EMBO Mol. Med. 5, 169–172 (2013). 23339072 10.1002/emmm.201202388 PMC3569633

[b6] Tomasetti C., Marchionni L., Nowak M. A., Parmigiani G. & Vogelstein B. Only three driver gene mutations are required for the development of lung and colorectal cancers. Proc. Natl Acad. Sci. USA 112, 118–123 (2015). 25535351 10.1073/pnas.1421839112 PMC4291633

[b7] Cancer Genome Atlas Research N. Comprehensive molecular characterization of gastric adenocarcinoma. Nature 513, 202–209 (2014). 25079317 10.1038/nature13480 PMC4170219

[b8] Cancer Genome Atlas Research Network. Comprehensive molecular profiling of lung adenocarcinoma. Nature 511, 543–550 (2014). 25079552 10.1038/nature13385 PMC4231481

[b9] Cancer Genome Atlas Network. Comprehensive molecular portraits of human breast tumours. Nature 490, 61–70 (2012). 23000897 10.1038/nature11412 PMC3465532

[b10] Cancer Genome Atlas Research Network. . Integrated genomic characterization of endometrial carcinoma. Nature 497, 67–73 (2013). 23636398 10.1038/nature12113 PMC3704730

[b11] Verhaak R. G. . Integrated genomic analysis identifies clinically relevant subtypes of glioblastoma characterized by abnormalities in PDGFRA, IDH1, EGFR, and NF1. Cancer Cell 17, 98–110 (2010). 20129251 10.1016/j.ccr.2009.12.020 PMC2818769

[b12] Simpson A. J., Caballero O. L., Jungbluth A., Chen Y. T. & Old L. J. Cancer/testis antigens, gametogenesis and cancer. Nat. Rev. Cancer 5, 615–625 (2005). 16034368 10.1038/nrc1669

[b13] Whitehurst A. W. Cause and consequence of cancer/testis antigen activation in cancer. Ann. Rev. Pharmacol. Toxicol. 54, 251–272 (2014). 24160706 10.1146/annurev-pharmtox-011112-140326

[b14] Coulie P. G. . Genes coding for tumor antigens recognized by human cytolytic T lymphocytes. J. Immunother. Emphasis Tumor Immunol. 14, 104–109 (1993). 8280701 10.1097/00002371-199308000-00004

[b15] De Backer O. . Characterization of the GAGE genes that are expressed in various human cancers and in normal testis. Cancer Res. 59, 3157–3165 (1999). 10397259

[b16] Boel P. . BAGE: a new gene encoding an antigen recognized on human melanomas by cytolytic T lymphocytes. Immunity 2, 167–175 (1995). 7895173 10.1016/s1074-7613(95)80053-0

[b17] Hofmann O. . Genome-wide analysis of cancer/testis gene expression. Proc. Natl Acad. Sci. USA 105, 20422–20427 (2008). 19088187 10.1073/pnas.0810777105 PMC2603434

[b18] Taguchi A. . A search for novel cancer/testis antigens in lung cancer identifies VCX/Y genes, expanding the repertoire of potential immunotherapeutic targets. Cancer Res. 74, 4694–4705 (2014). 24970476 10.1158/0008-5472.CAN-13-3725 PMC4398029

[b19] Almeida L. G. . CTdatabase: a knowledge-base of high-throughput and curated data on cancer-testis antigens. Nucleic Acids Res. 37, D816–D819 (2009). 18838390 10.1093/nar/gkn673 PMC2686577

[b20] Madison B. B. . LIN28B promotes growth and tumorigenesis of the intestinal epithelium via Let-7. Genes Dev. 27, 2233–2245 (2013). 24142874 10.1101/gad.224659.113 PMC3814644

[b21] Molenaar J. J. . LIN28B induces neuroblastoma and enhances MYCN levels via let-7 suppression. Nat. Genet. 44, 1199–1206 (2012). 23042116 10.1038/ng.2436

[b22] Piskounova E. . Lin28A and Lin28B inhibit let-7 microRNA biogenesis by distinct mechanisms. Cell 147, 1066–1079 (2011). 22118463 10.1016/j.cell.2011.10.039 PMC3227872

[b23] Viswanathan S. R. . Lin28 promotes transformation and is associated with advanced human malignancies. Nat. Genet. 41, 843–848 (2009). 19483683 10.1038/ng.392 PMC2757943

[b24] Zhou J., Ng S. B. & Chng W. J. LIN28/LIN28B: an emerging oncogenic driver in cancer stem cells. Int. J. Biochem. Cell Biol. 45, 973–978 (2013). 23420006 10.1016/j.biocel.2013.02.006

[b25] Matzuk M. M. . Small-molecule inhibition of BRDT for male contraception. Cell 150, 673–684 (2012). 22901802 10.1016/j.cell.2012.06.045 PMC3420011

[b26] GT Consortium. Human genomics. The Genotype-Tissue Expression (GTEx) pilot analysis: multitissue gene regulation in humans. Science 348, 648–660 (2015). 25954001 10.1126/science.1262110 PMC4547484

[b27] Mele M. . Human genomics. The human transcriptome across tissues and individuals. Science 348, 660–665 (2015). 25954002 10.1126/science.aaa0355 PMC4547472

[b28] Rivas M. A. . Human genomics. Effect of predicted protein-truncating genetic variants on the human transcriptome. Science 348, 666–669 (2015). 25954003 10.1126/science.1261877 PMC4537935

[b29] Cancer Genome Atlas Research Network. . The Cancer Genome Atlas Pan-Cancer analysis project. Nat Genet. 45, 1113–1120 (2013). 24071849 10.1038/ng.2764 PMC3919969

[b30] Kim M. S. . A draft map of the human proteome. Nature 509, 575–581 (2014). 24870542 10.1038/nature13302 PMC4403737

[b31] Wang Z., Gerstein M. & Snyder M. RNA-Seq: a revolutionary tool for transcriptomics. Nat. Rev. Genet. 10, 57–63 (2009). 19015660 10.1038/nrg2484 PMC2949280

[b32] Huang, da W., Sherman B. T. & Lempicki R. A. Systematic and integrative analysis of large gene lists using DAVID bioinformatics resources. Nat. Protoc. 4, 44–57 (2009). 19131956 10.1038/nprot.2008.211

[b33] Park C., Yu N., Choi I., Kim W. & Lee S. lncRNAtor: a comprehensive resource for functional investigation of long non-coding RNAs. Bioinformatics 30, 2480–2485 (2014). 24813212 10.1093/bioinformatics/btu325

[b34] Iyer M. K. . The landscape of long noncoding RNAs in the human transcriptome. Nat. Genet. 47, 199–208 (2015). 25599403 10.1038/ng.3192 PMC4417758

[b35] Harada Y. . Cell-permeable peptide DEPDC1-ZNF224 interferes with transcriptional repression and oncogenicity in bladder cancer cells. Cancer Res. 70, 5829–5839 (2010). 20587513 10.1158/0008-5472.CAN-10-0255

[b36] Wawrzik M., Spiess A. N., Herrmann R., Buiting K. & Horsthemke B. Expression of SNURF-SNRPN upstream transcripts and epigenetic regulatory genes during human spermatogenesis. Eur. J. Hum. Genet. 17, 1463–1470 (2009). 19471314 10.1038/ejhg.2009.83 PMC2986690

[b37] Luo M. . MEIOB exhibits single-stranded DNA-binding and exonuclease activities and is essential for meiotic recombination. Nat. Commun. 4, 2788 (2013). 24240703 10.1038/ncomms3788 PMC3891831

[b38] Watkins J. . Genomic complexity profiling reveals That HORMAD1 overexpression contributes to homologous recombination deficiency in triple-negative breast cancers. Cancer Discov. 5, 488–505 (2015). 25770156 10.1158/2159-8290.CD-14-1092 PMC4490184

[b39] Ogi C. & Aruga A. Immunological monitoring of anticancer vaccines in clinical trials. Oncoimmunology 2, e26012 (2013). 24083085 10.4161/onci.26012 PMC3782518

[b40] Ulloa-Montoya F. . Predictive gene signature in MAGE-A3 antigen-specific cancer immunotherapy. J. Clin. Oncol. 31, 2388–2395 (2013). 23715562 10.1200/JCO.2012.44.3762

[b41] Vansteenkiste J. . Adjuvant MAGE-A3 immunotherapy in resected non-small-cell lung cancer: phase II randomized study results. J. Clin. Oncol. 31, 2396–2403 (2013). 23715567 10.1200/JCO.2012.43.7103

[b42] Huang M., Shen A., Ding J. & Geng M. Molecularly targeted cancer therapy: some lessons from the past decade. Trends Pharmacol. Sci. 35, 41–50 (2014). 24361003 10.1016/j.tips.2013.11.004

[b43] Vanneman M. & Dranoff G. Combining immunotherapy and targeted therapies in cancer treatment. Nat Rev. Cancer 12, 237–251 (2012). 22437869 10.1038/nrc3237 PMC3967236

[b44] Pineda C. T. . Degradation of AMPK by a cancer-specific ubiquitin ligase. Cell 160, 715–728 (2015). 25679763 10.1016/j.cell.2015.01.034 PMC5629913

[b45] Baudat F., Imai Y. & de Massy B. Meiotic recombination in mammals: localization and regulation. Nat. Rev. Genet. 14, 794–806 (2013). 24136506 10.1038/nrg3573

[b46] Orthwein A. . Mitosis inhibits DNA double-strand break repair to guard against telomere fusions. Science 344, 189–193 (2014). 24652939 10.1126/science.1248024

[b47] Fratta E. . The biology of cancer testis antigens: putative function, regulation and therapeutic potential. Mol. Oncol. 5, 164–182 (2011). 21376678 10.1016/j.molonc.2011.02.001 PMC5528287

[b48] Assenov Y. . Comprehensive analysis of DNA methylation data with RnBeads. Nat. Methods 11, 1138–1140 (2014). 25262207 10.1038/nmeth.3115 PMC4216143

[b49] Van Loo P. . Allele-specific copy number analysis of tumors. Proc. Natl Acad. Sci. USA 107, 16910–16915 (2010). 20837533 10.1073/pnas.1009843107 PMC2947907

[b50] Bullard J. H., Purdom E., Hansen K. D. & Dudoit S. Evaluation of statistical methods for normalization and differential expression in mRNA-Seq experiments. BMC Bioinformatics 11, 94 (2010). 20167110 10.1186/1471-2105-11-94 PMC2838869

