# Supplementary material for: Systematic identification of genes with a cancer-testis expression pattern in 19 cancer types
Source: Nat Commun. 2016 Jan 27;7:10499. doi: 10.1038/ncomms10499 (PMC4737856; doi:10.1038/ncomms10499)
Supplement: Supplementary Information — Supplementary Figures 1-9, Supplementary Tables 1-2, Supplementary Methods and Supplementary References. [file ncomms10499-s1.pdf]

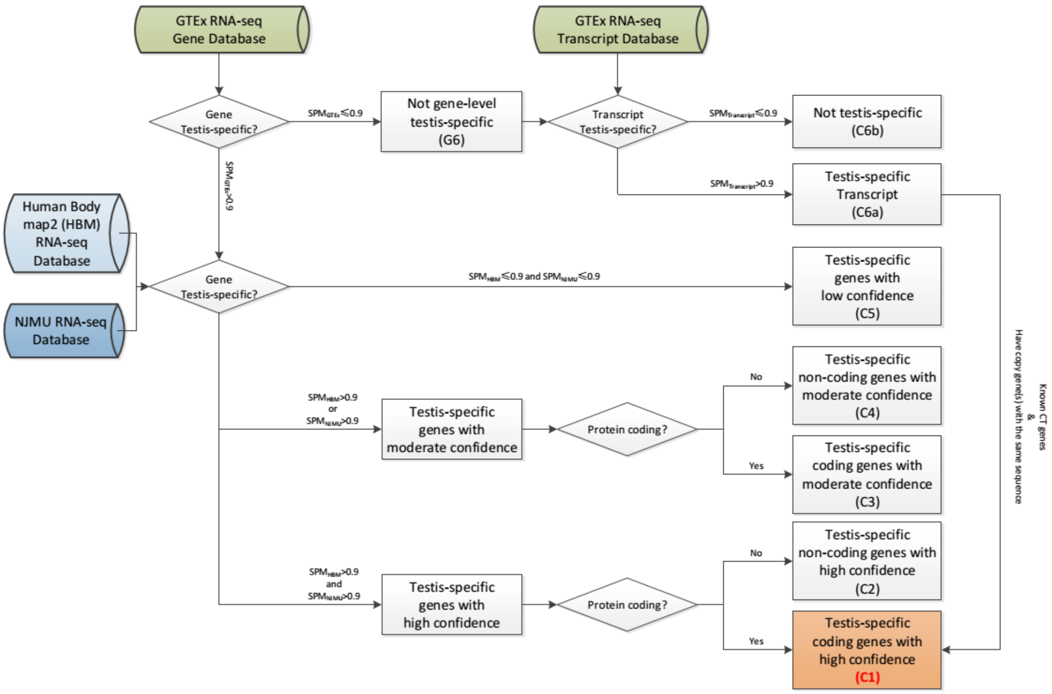

Supplementary Figure 1. General strategy to classify genes and identify TSGs.

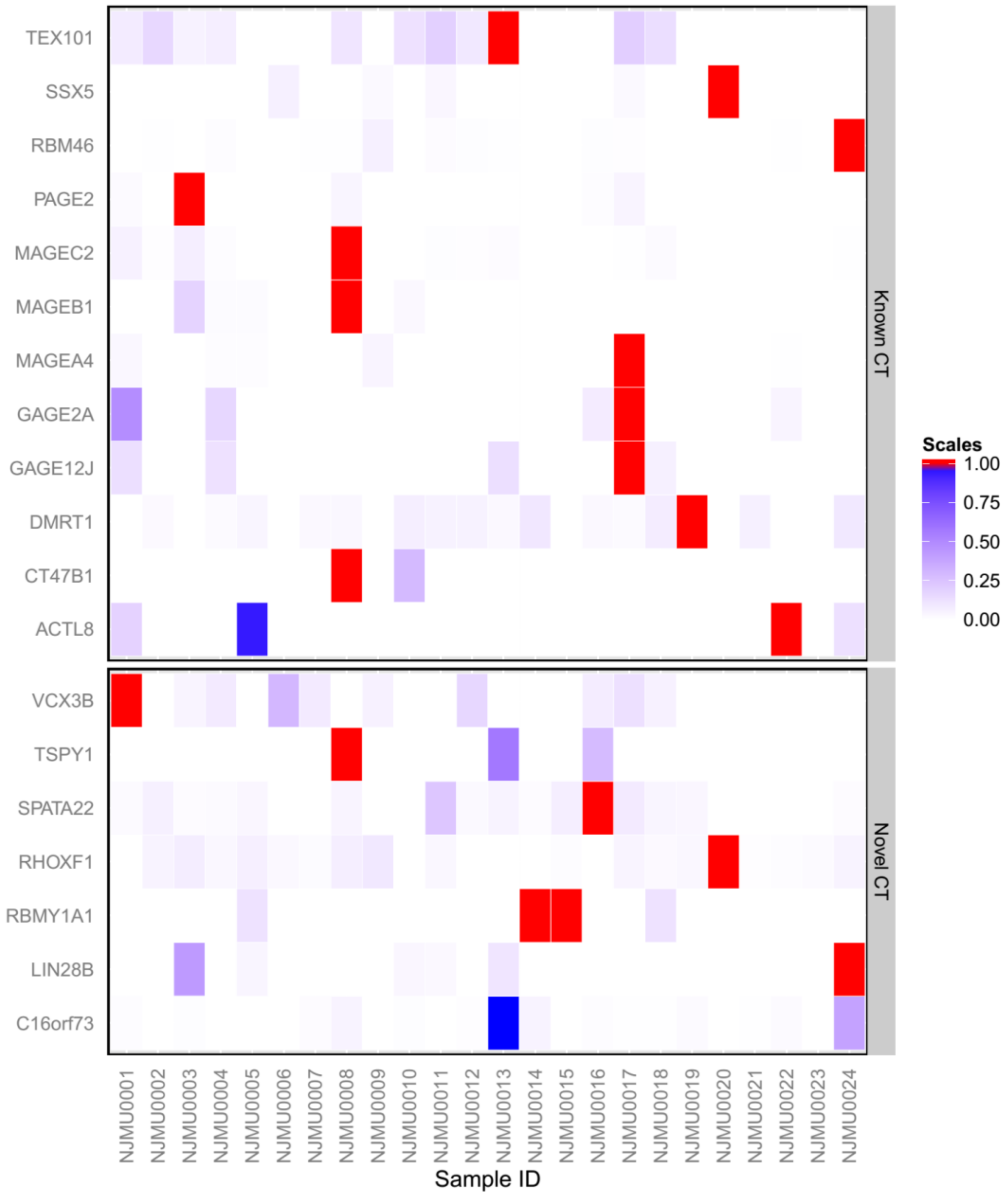

Supplementary Figure 2. EE patterns of validated EECTPs (7 novel) in our 24 LUAD samples.

Red indicates extremely highly expressed samples, and blue indicates other samples. The depth of color indicates the degree of expression level. Because the expression of *MEIOB* of sample 130717001 approaches the extremely-high expression criteria and its co-factor *SPATA22* is validated, we consider it as a validated EECTP and include it in the further functional assay.

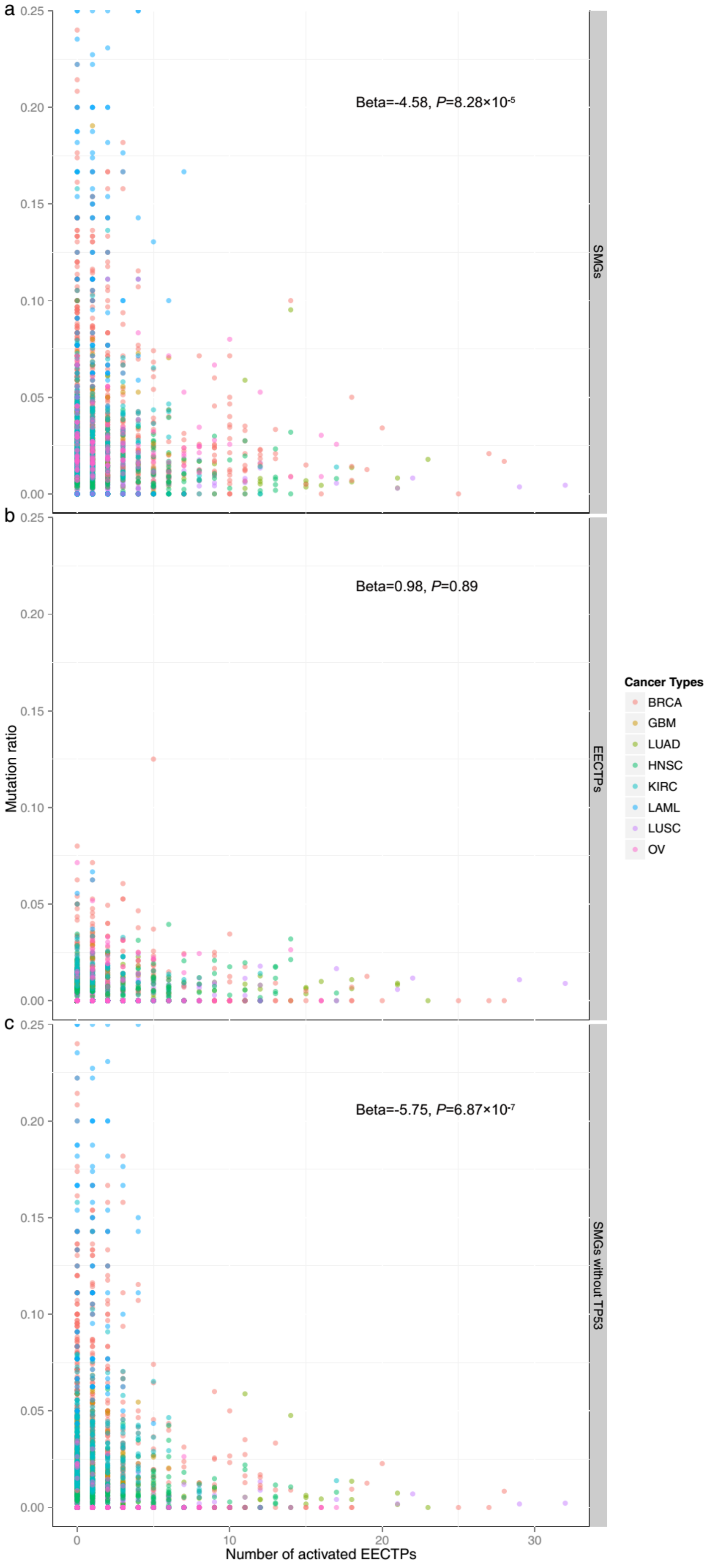
a
b
c
Beta=-4.58, P=8.28×10-5
Beta=0.98, P=0.89
Beta=-5.75, P=6.87×10-7
SMGs
EECTPs
SMGs without TP53
Mutation ratio
Number of activated EECTPs
Cancer Types
BRCA
GBM
LUAD
HNSC
KIRC
LAML
LUSC
OV
0.25
0.20
0.15
0.10
0.05
0.00
0
10
20
30

Supplementary Figure 3. The association between SMG mutation ratio and number of activated EECTPs.

(a) The association of SMG mutation ratio and the number of activated EECTPs.

(b) The association of EECTPs mutation ratio and the number of activated EECTPs.

(c) The association of SMG mutation ratio (exclude mutations in *TP53*) and the number of activated EECTPs.

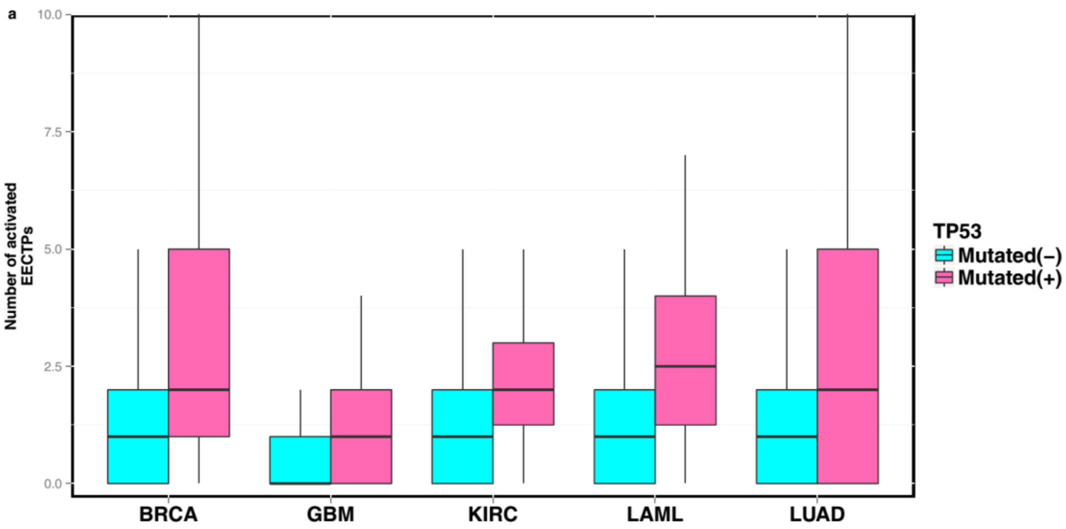
a
Number of activated EECTPs
10.0
7.5
5.0
2.5
0.0
BRCA
GBM
KIRC
LAML
LUAD
TP53
Mutated(−)
Mutated(+)

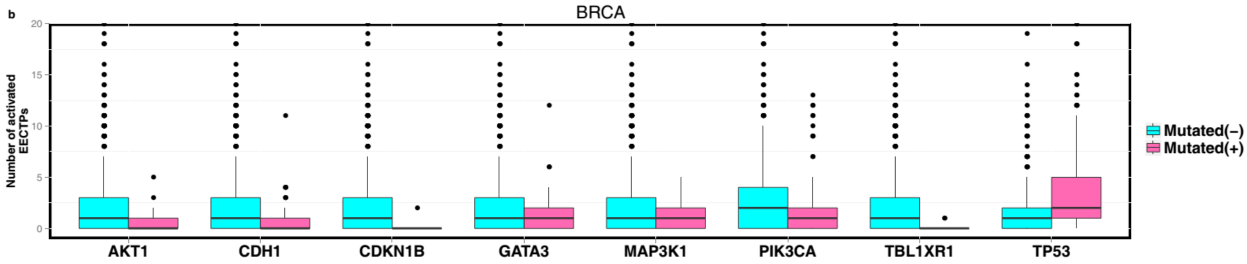
b
BRCA
Number of activated EECTPs
20
15
10
5
0
AKT1
CDH1
CDKN1B
GATA3
MAP3K1
PIK3CA
TBL1XR1
TP53
Mutated(−)
Mutated(+)

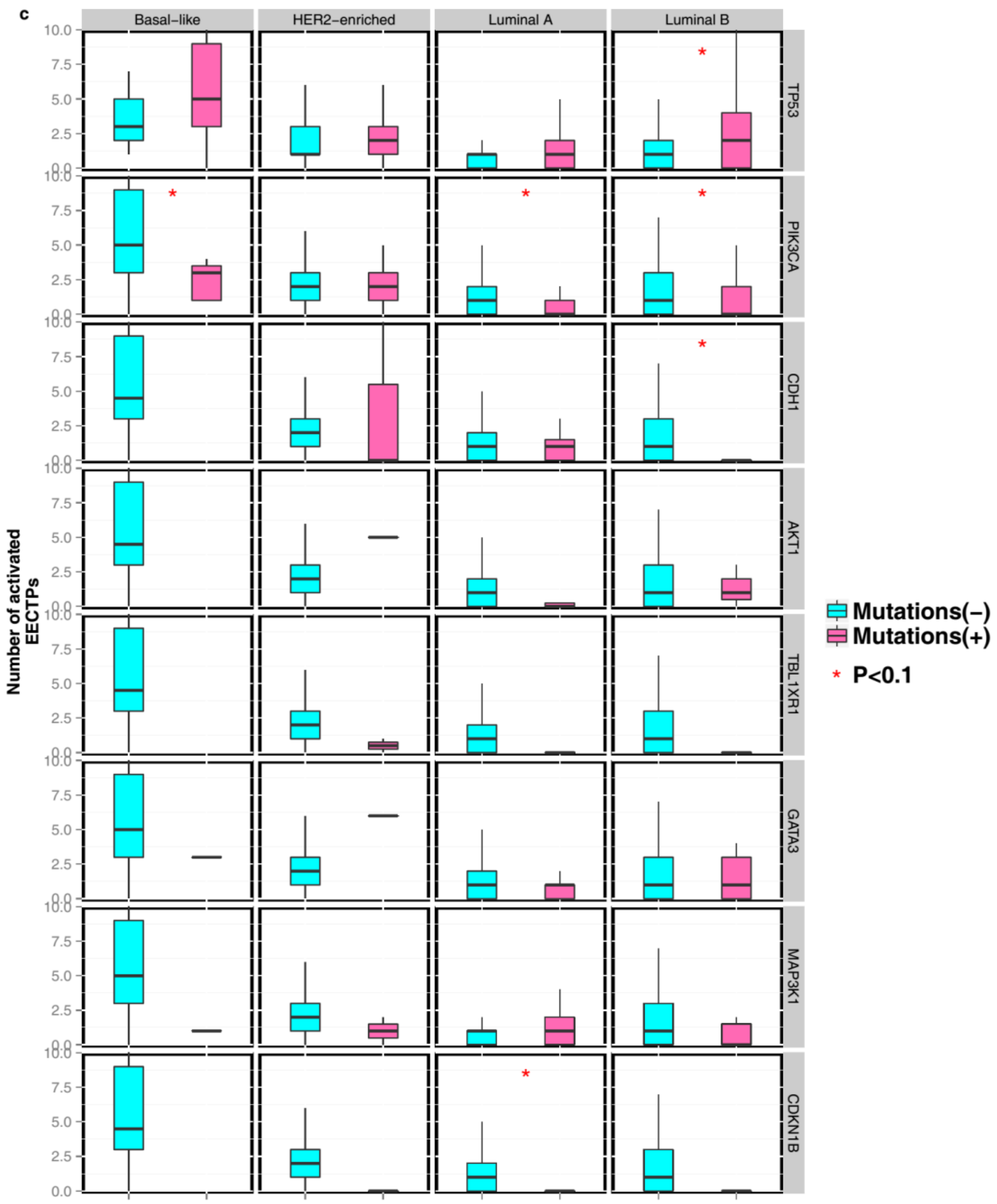

Supplementary Figure 4. SMGs in which mutations are significantly associated with the number of activated EECTPs.

(a) The number of activated EECTPs is significantly higher in patients with TP53 mutations in multiple cancers.

(b) BRCA SMGs in which mutations are significantly associated with the number of

activated EECTPs.

(c) *PIK3CA* is consistently associated with the activation of EECTPs in multiple molecular subtypes of BRCA.

The box plot displays the first and third quartiles (top and bottom of the boxes), the median (band inside the boxes), and the lowest and highest point within 1.5 times the interquartile range of the lower and higher quartile (whiskers).

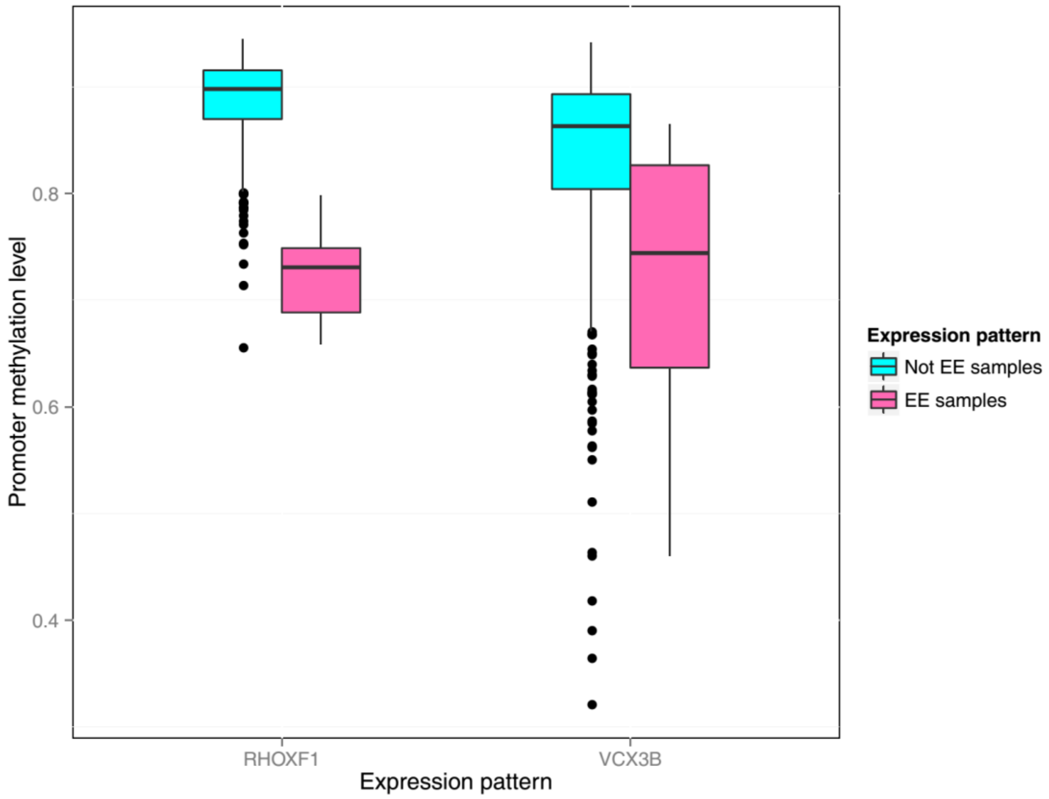

Supplementary Figure 5. Negative correlation between promoter methylation level and activated expression of *RHOXF1* and *VCX3B*.

The box plot displays the first and third quartiles (top and bottom of the boxes), the median (band inside the boxes), and the lowest and highest point within 1.5 times the interquartile range of the lower and higher quartile (whiskers).

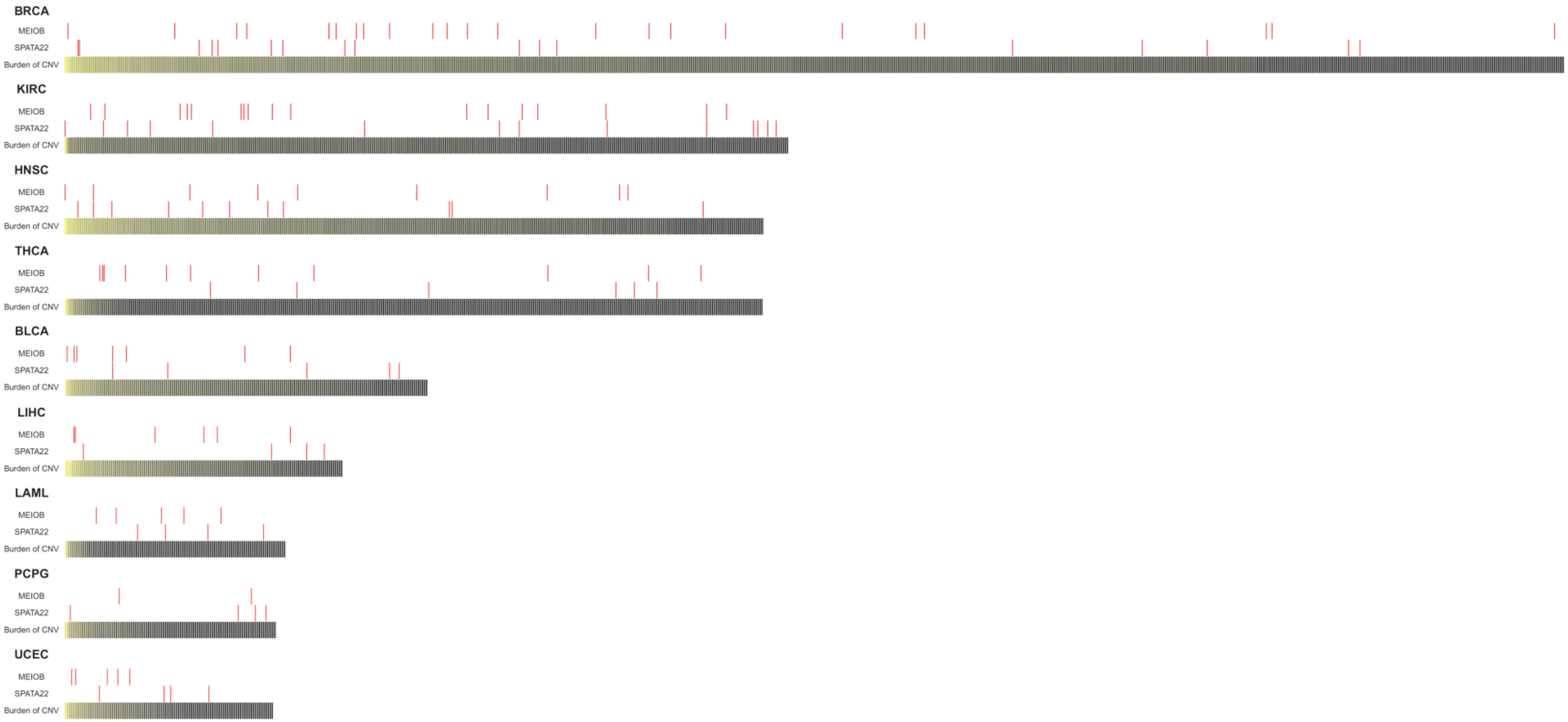

Supplementary Figure 6. Mutually exclusive EE patterns of *MEIOB* and *SPATA22* in other tumor types with *MEIOB* or *SPATA22* activation.

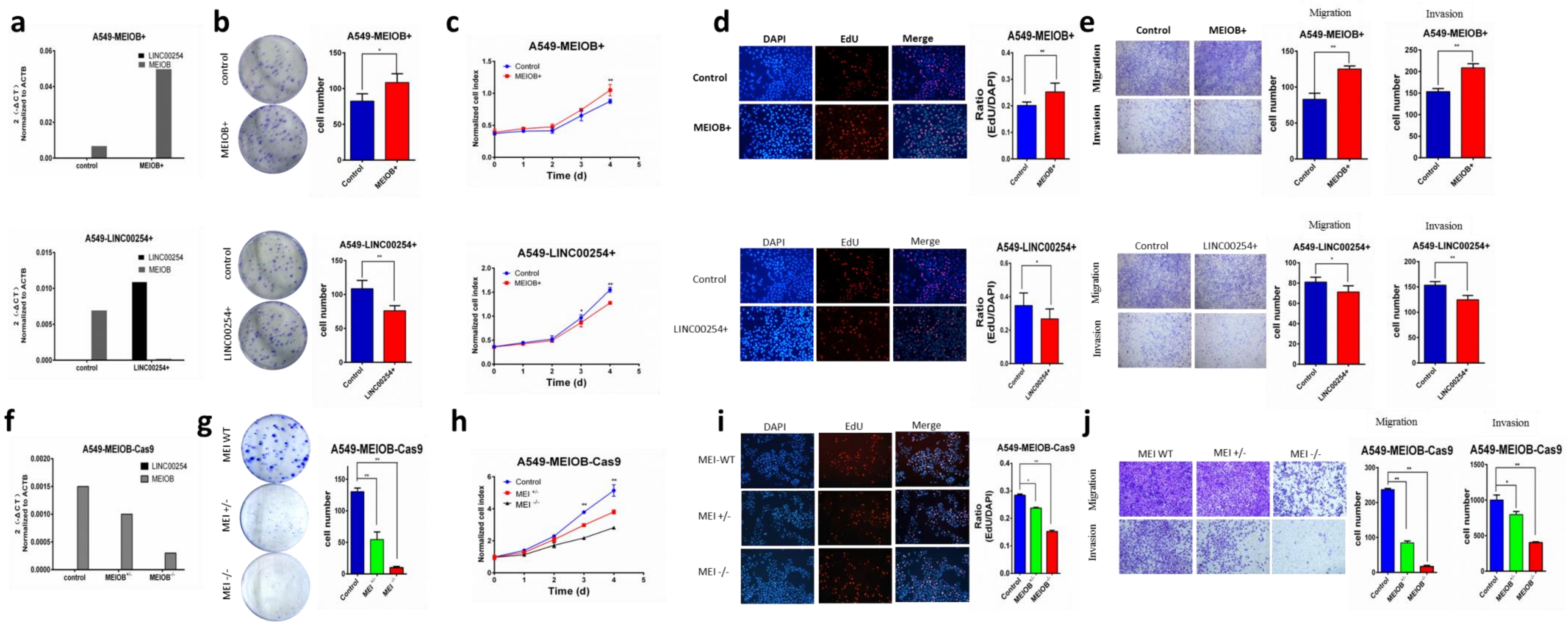

Supplementary Figure 7. Detailed results of the functional assay of *MEIOB/LINC00254* in A549 cell lines.

(a) and (f). Relative expression of *MEIOB* and *LINC00254* in the differently treated A549 cells.

(b-e). Overexpression of *MEIOB* promoted A549 growth (colony formation, growth curve and EdU staining), migration and invasion. Overexpression of *LINC00254* led to the opposite results.

(g-j). Knockout *MEIOB* reduced growth, migration and invasion of A549 cells.

Error bars represent s.e.m, n=5. * represent $P<0.05$ compared with the vector control. ** represent $P<0.001$ compared with the vector control. All of the experiments were repeated three times.

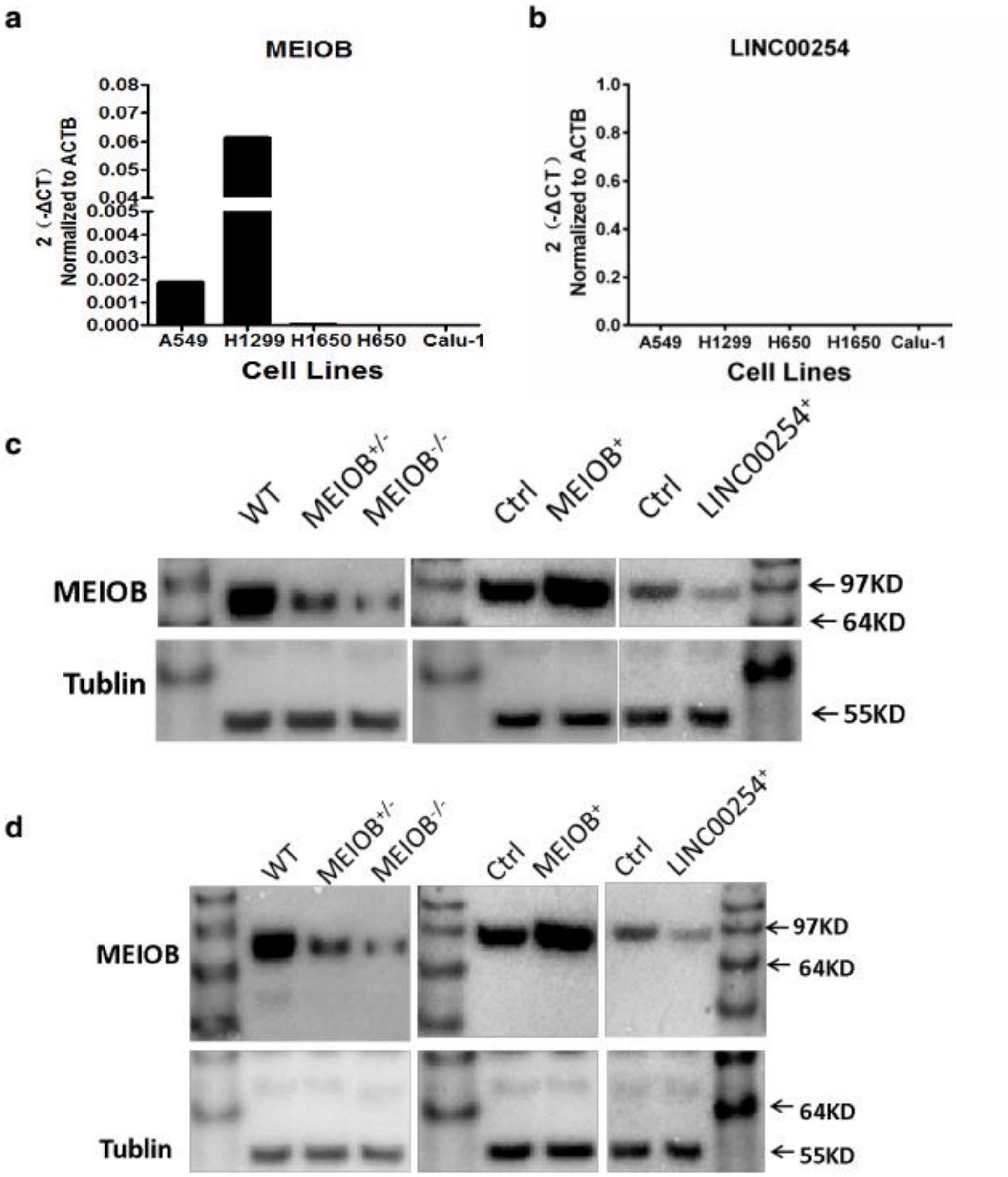

Supplementary Figure 8. Relative expression of *MEIOB*/*LINC00254* in lung cancer cell lines and protein blot of A549 cells knocked out or overexpressed with *MEIOB*/*LINC00254*.

(a) Relative expression of *MEIOB* in lung cancer cell lines.

(b) Relative expression of *LINC00254* in lung cancer cell lines.

(c) Overexpression of *LINC00254* reduced the expression of *MEIOB* in A549 cells.

(d) Uncropped scans of western blots in (c).

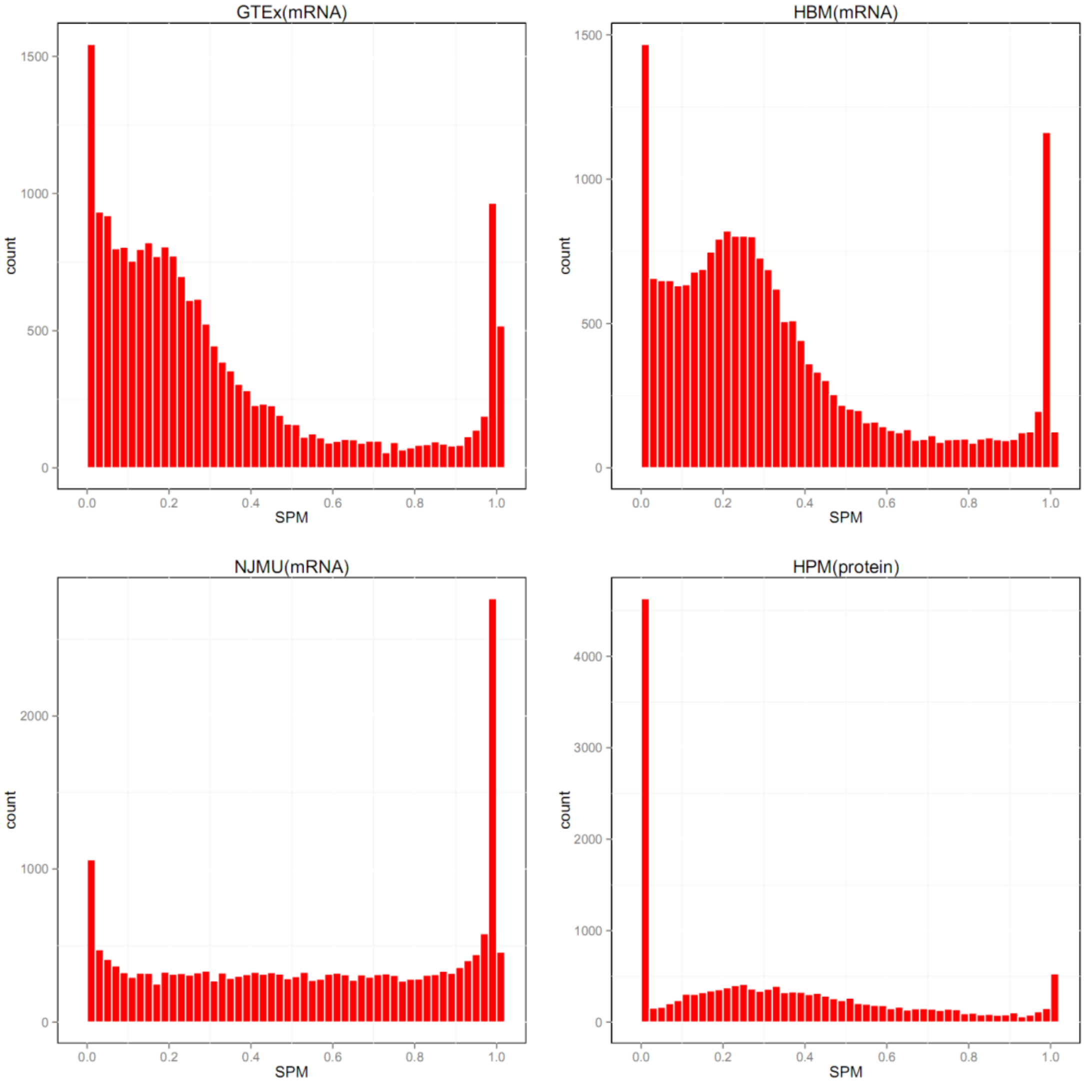

Supplementary Figure 9. Distribution of SPM values Calculated from each database.

Supplementary Tables

Supplementary Table 1. Public datasets used in this study. Detailed summary of samples was listed in the Supplementary Data 1.

Database Name	Version or Web Sites	Platform	Data Type	Sample Type	Sample Size
GTEx project	phs000424.v3.p1	Illumina HiSeq/GAII RNA-seq	Processed (FPKM)	Normal	175
Illumina Human Body Map 2.0	E-MTAB-513	Illumina HiSeq RNA-seq	Raw (Fastq)	Normal	14
Human protein map	http://humanproteomemap.org/	LC-MS/MS	Processed (Normalized spectral counts)	Normal	16
Fantom	release 5	Cap Analysis of Gene Expression	Processed (Expression matrix)	Normal	38
Encode	Mar 2012 Freeze	Reduced Representation Bisulfite Seq	Processed (Sites)	Normal	15
TCGA	Broad GDAC Firehose (2014-07-15)	Illumina HiSeq RNA sequencing	Processed (RSEM)	Tumor	6638
TCGA	Pancan (syn1729383)	Illumina HiSeq whole-exome sequencing	Processed (MAF)	Tumor	2315
TCGA	TCGA data portal	Illumina Infinium HumanMethylation450 BeadChip	Raw (idat)	Tumor	2682
TCGA	TCGA data portal	Affymetrix SNP6.0 array	Processed (focal CNV) & Raw (CEL, LUAD)	Tumor	3973
mitranscriptome	http://www.mitranscriptome.org	Illumina HiSeq RNA sequencing	Processed (normalized read counts)	Tumor	-
lncrnator	http://lncrnator.ewha.ac.kr/index.htm	Illumina HiSeq RNA sequencing	Processed (FPKM)	Tumor	-

Supplementary Table 2A. Sequences of sgRNAs

Gene name	sgRNA sequence(5'-3')
MEIOB	5'-ATGCGTCTCAACCGTCTCTTTCTGACAGCTTTAGTTTTAGAGCTAGAAATAGCAAG(forward)
	5'-ATGCGTCTCGAAACGCACATTTTGTAAATGCAGCGGTGTTTCGTCCTTTCCACAAG(reverse)
LINC00254	5'-ATGCGTCTCGAAACACCGCCCATCAGGTTGTTTCGGTGTTTCGTCCTTTCCACAAG(forward)
	5'-ATGCGTCTCAACCGAAAATAAATGGGGTTTAGGGTTTTAGAGCTAGAAATAGCAAG(reverse)

Supplementary Table 2B. Primers for amplifying sgRNA target site and sequencing

Gene name	Primer sequence(5'-3')	Amplicon (bp)
MEIOB	5'-GCAACCTGTTACCACTTCA(forward)	481bp
	5'-CTTGAGAATTACGAACTGTGTC(reverse)	
hL254 sg12	5'-CTCCATATCAACTCCACATTAC(forward)	421bp
	5'-GGAATCACTGTTGTGACATT(reverse)	
hL254 sg34	5'-GTACCAATCTGCCAGTCT(forward)	651bp
	5'-TCAGAGCTTGAGAACCTATT(reverse)	

Supplementary Methods

mRNA expression quantification in GTEx and Illumina Human Body Map 2.0

Gene and transcript expression profiles (evaluated by FPKM value) of 24 types of normal tissues collected from 175 samples were downloaded from the GTEx data portal (http://www.gtexportal.org/, GTEx Analysis Pilot Data 2013-01-31, dbGaP Accession phs000424.v3.p1, Supplementary Table 1). We used the median FPKM of each tissue for SPM calculation.

To estimate the expression of the non-coding RNAs (ncRNAs) in Illumina Human Body Map (HBM) and compare with the ncRNAs with testis-specific expression patterns (TS-ncRNAs) identified by the GTEx, we downloaded raw FASTQ files from E-MTAB-513 and performed a comprehensive RNA-seq analysis on 14 normal tissues from HBM. Initial sequence quality was evaluated using FASTQC (http://www.bioinformatics.babraham.ac.uk/projects/fastqc). Cutadapt (http://code.google.com/p/cutadapt/) was used to trim Illumina sequencing adaptors and poor-quality bases with a quality score of 20 and discard reads with a length below 30 bp after trimming. Reads were mapped to the reference genome (GENCODE Version 19, http://www.gencodegenes.org/releases/19.html) using TopHat2 [1] (v2.0.9) with default parameters. Reference genome annotation files and the transcriptome reference gene set were downloaded from the GENCODE v19 databases. Cufflinks [2] (v2.2.1) was used to assemble transcripts and to estimate expression abundances with the parameter "-G."

Database combination and gene annotation

In this study, ENSEMBL ID from GENCODE v19 was regarded as the official indicator for further analysis. All databases annotated by other references were re-annotated by an R package biomaRt[3, 4]. Any genes/proteins that failed to annotate unambiguously were excluded from the subsequent analysis.

Methods to evaluate testis-specific genes (TSGs)

In this study, the specificity measure [5] (SPM) was used to evaluate the testis-specific expression pattern.

Each gene expression profile is transformed into a vector :

$$X = (x_1, x_2, \dots, x_n, x_{testis}) \quad [1]$$

where n is the number of tissues in the profile. Similarly, a vector X can be generated to represent the gene expression in testis:

$$X_{testis} = (0, 0, \dots, 0, x_{testis}) \quad [2]$$

SPM is the cosine value of the intersection angle θ between vectors X_{testis} and X in high dimensional feature space. This variable is calculated by the following expression:

$$SPM = \cos\theta = \frac{X_{testis} \cdot X}{|X_{testis}| \cdot |X|} \quad [3]$$

where $|X_{testis}|$ and $|X|$ are the length of vectors X_{testis} and X, respectively. SPM values range from 0 to 1, with values close to 1 indicating a major contribution to gene expression in a testis (vector X_{testis}) relative to all other tissues (vector X). Testis-specific genes were defined as genes with SPM higher than 0.9, thus including both testis-restricted and testis-selective genes.

Protein expression quantification in human protein map (HPM)

Normalized spectral counts data were downloaded from http://www.humanproteomemap.org/download.php. Because the SPM distribution calculated from protein spectral counts was similar to the SPM distribution calculated from mRNA abundance (Supplementary Figure 9), we chose the same cutoff (0.9) to identify testis-specific proteins (TSPs).

Enrichment analysis of testis-specific regulatory elements (TSREs)

In this study, we performed enrichment analysis to evaluate the relationship between the TSREs and the TSGs. Four types of regulatory elements were included in the analysis (promoter, methylation level, ncRNA and enhancer).

Genes from C2 and C4 groups were considered as testis-specific non-coding RNAs (TS-ncRNAs) in our analysis. To avoid ambiguous mapping which derived from overlapping exons of protein-coding genes, we excluded ncRNA that overlapped with the exons of protein-coding genes in the same strand.

The activity of promoters and enhancers was estimated by Cap Analysis of Gene Expression

(CAGE) from the Fantom project[6]. We downloaded CAGE expression levels (http://fantom.gsc.riken.jp/data/) to reflect the activity of regulatory elements and calculated SPM values for each promoter/enhancer to identify testis-specific activity (SPM cutoff: 0.9). Beta values were downloaded from human Reduced Representation Bisulfite Sequencing (RRBS) of the ENCODE project (http://genome.ucsc.edu/ENCODE/) to evaluate methylation level. Only sites with coverage greater than 5 were used for evaluation. Thus, 577,925 sites had beta values in all 15 samples and were used for the definition of the testis-specific methylation site (TSMS). Because of the bimodal distribution of beta value, we could not apply the SPM method. In our study, we defined sites with 25% lower beta value[7, 8] in the testis than in other normal tissues as TSMS.

mRNA expression quantification in TCGA data

We obtained level 3-normalized TCGA RNA-seqV2 expression quantification data from Firehose at the MIT Broad Institute (https://confluence.broadinstitute.org/display/GDAC/Home, 2014-07-15 release). Twenty cancer types with more than 100 samples were included in the identification of CT genes. Gene expression was quantified for the transcript models corresponding to the TCGA GAF2.1 using RSEM and normalized within sample to a fixed upper quartile. When defining extremely highly expressed (EE) patterns, expression values of zero were set to one and all data were log2 transformed.

Sample preparation and mRNA expression quantification in the NJMU lung adenocarcinoma (LUAD) tumor/normal data

To validate the extremely high expression (EE) patterns of identified EECTPs, we performed RNA sequencing using Illumina sequencing technology on poly(A)-selected RNA from 24 lung adenocarcinoma samples and their adjacent normal tissues in the NJMU study. Samples were collected from Affiliated Hospitals of Nanjing Medical University. Tissues samples were preserved using RNA-later solution. HE-stained sections from each sample were subjected to independent pathology review to confirm that the tumor specimen was histologically consistent with LUAD (>70% tumor cells) and that the adjacent tissue

specimen contained no tumor cells. Total RNA was extracted from cell lines and tissue samples using the RNeasy Mini Kit (Qiagen, Hilden, Germany) according to the manufacturer's instructions. The extracted RNA samples were analyzed using an Agilent 2100 Bioanalyzer system (Agilent Biotechnologies, Palo Alto, USA) with the RNA 6000 Nano Labchip Kit. Only samples of high-quality RNA (RNA Integrity Number≥7.5) were used in the subsequent mRNA sample preparation for sequencing. PolyA-minus RNAs were fractionated from total RNA samples and RNA-seq libraries were generated by RNA-fragmentation, random hexamer-primed cDNA synthesis, linker ligation and PCR amplification using a TruSeq™ RNA Sample Prep Kit (Illumina, Inc.). The purified DNA libraries were sequenced with Illumina HiSeq1500 platform (paired-end, 100 base).

Quality control processes followed the same protocols for handling RNA-seq data in normal tissues. Gene expression was quantified for the transcript models corresponding to the GENCODE v19 using RSEM and normalized within sample to a fixed upper quartile.

The definition of EE and activated EECTG/Ps

For each EECTG/EECTP, all samples were classified as activated samples or inactivated samples based on whether their expression exceeded the extremely high expression cutoff ($Mean_{\log_2 Normalized\ Counts} + 3 \times SD_{\log_2 Normalized\ Counts}$) and were recoded as 1 and 0 respectively. For each sample, number of activated EECTPs (count of EECTPs which were coded as 1) was used to represent the degree driven by EECTPs. In our LUAD validation, because the expression of *MEIOB* of sample 130717001 approaches the extremely-high expression criteria and its co-factor *SPATA22* is validated, we consider it as a validated EECTP and include it in the further functional assay.

Obtaining and processing somatic mutation data sets

As described in the result sections, we obtained somatic mutation information to explore the relationship between the EE pattern of EECTPs and somatic mutations. Mutation data were downloaded from the Synapse platform (syn1729383) as “maf” files within the context of the PANCANCER project. Only cancer types with more than 100 samples with both expression and mutation data were included in the analysis, and EECTPs were redefined using data of

platform overlapped samples. Impact scores given by the IntOGen-mutations Web discovery tool (http://www.intogen.org/search) were used to evaluate the potential functions of mutations.

Significantly mutated genes (SMGs) of each cancer were obtained from Supplementary Table 4 of a previously published paper[9]. The mutation ratio represented the degree of samples driven by SMG mutations and was calculated as the ratio of the mutation number in SMGs and the mutation number in all genes. Driver summary of papillary thyroid carcinoma were obtained from the Supplementary Table 2 of previous study[10].

Linear regression was used to evaluate the association between the mutation ratio and activated number of EECTPs. For each SMG, a Wilcoxon's rank sum test was used for the statistical comparison of the activated EECTP number between mutated and non-mutated samples. Fisher's exact test was employed to test mutually exclusive patterns between the SMGs' mutations and EECTPs' EE patterns. *P*-values were adjusted by Benjamini–Hochberg false discovery rate (FDR-BH). PAM50 subtypes were obtained from the related data from a previous paper [11].

Obtaining and processing methylation data sets

Seven cancer types had more than 100 samples with both expression and methylation data were included in the analysis, and EECTPs were redefined using data of platform overlapped samples. We downloaded Illumina raw idat-files produced by the Infinium HumanMethylation450 BeadChip Kit of these cancer types from the TCGA data portal (https://tcga-data.nci.nih.gov/tcga/tcgaHome2.jsp) and called the methylation levels (beta value). The default RnBeads [12] workflow was executed by running the rnb.run.analysis (...) command to perform quality control and preprocessing module. Because many CT genes are located in the sex chromosomes, methylation data in sex chromosomes were kept by using rnb.options to set global configuration parameters. We used beta values of the promoters from the output of RnBeads for further analysis. Mean beta values were used to evaluate the methylation level of multiple sites or regions.

Linear regression was used to evaluate the association between the average promoter

methylation level of EECTPs and the activated number EECTPs. For each EECTP, a Wilcoxon's rank sum test was used for statistical comparison of methylation levels between activated and inactivated samples. *P*-values were adjusted by Benjamini–Hochberg false discovery rate (FDR-BH).

The definition of CT-ncRNA and data processing

We downloaded the expression quantification of differential expressed ncNRAs from lncrnator [13] and the expression quantification of cancer/lineage associated ncNRAs from Mitranscriptome [14]. Because Mitranscriptome groups conducted de-novo assembly of ncRNAs, we annotated Mitranscriptome transcripts with GENCODE v19 according to the coordinates of transcripts. A Mitranscriptome transcript was successfully annotated if it overlapped with any GENCODE transcript and the proportion of overlapped region and transcript length of GENCODE was greater than 80%.

Spearman's rank correlation test was used to estimate the correlation coefficient of the expression of CT-ncRNAs and nearby protein-coding CT genes. The cancer types were included in the correlation analysis which had more than ten samples with both expression of CT coding genes and CT-ncRNAs. *P*-values were adjusted by Benjamini–Hochberg false discovery rate (FDR-BH).

Obtain and processing copy number data

We obtained level 3-focal copy number data from Firehose at the MIT Broad Institute (https://confluence.broadinstitute.org/display/GDAC/Home, 2014-07-15 release). Ten cancer types with *MEIOB* and *SPATA22* activation were included in this analysis (Supplementary Data 1).

For allele specific copy number analysis, raw .CEL files from genome-wide SNP6.0 microarray data of LUAD samples were preprocessed by R package affy2sv[15] and allele-specific copy number profiling was performed with ASCAT v2.1[16].

Scores of chromosomal instability scarring (SCINS) were calculated using the following steps of previous study[17]:

1) The proportion of the genome consisting of AiCNA segments, save those segments that

encompass a whole chromosome, is calculated.

2) The number of AiCNA segments greater than or equal to 8Mb in length but less than the length of a whole chromosome is counted.

3) The measure of AiCNA segments (S_{AiCNA}) is calculated by multiplying the proportion obtained in step 1) by the number of segments counted in step 2).

4) The proportion of the genome consisting of CnLOH segments is calculated.

5) The number of CnLOH segments greater than or equal to 4Mb in length, including those that span a whole chromosome, is counted.

6) The measure of CnLOH segments (S_{CnLOH}) is calculated by multiplying the proportion obtained in step 4) by the number of segments counted in step 5).

7) The measure of AbCNA segments (S_{AbCNA}) is calculated by counting the number of AbCNA segments greater than or equal to 8Mb in length.

8) The measure of all allelic imbalanced segments (S_{Ai}) is calculated by summing S_{AiCNA} and S_{CnLOH}.

Supplementary References

1. Trapnell C, Pachter L, Salzberg SL. TopHat: discovering splice junctions with RNA-Seq. *Bioinformatics* **25**, 1105-1111 (2009).

2. Trapnell C*, et al.* Differential gene and transcript expression analysis of RNA-seq experiments with TopHat and Cufflinks. *Nature protocols* **7**, 562-578 (2012).

3. Durinck S, Spellman PT, Birney E, Huber W. Mapping identifiers for the integration of genomic datasets with the R/Bioconductor package biomaRt. *Nature protocols* **4**, 1184-1191 (2009).

4. Durinck S*, et al.* BioMart and Bioconductor: a powerful link between biological databases and microarray data analysis. *Bioinformatics* **21**, 3439-3440 (2005).

5. Xiao SJ, Zhang C, Zou Q, Ji ZL. TiSGeD: a database for tissue-specific genes. *Bioinformatics* **26**, 1273-1275 (2010).

6. Consortium F*, et al.* A promoter-level mammalian expression atlas. *Nature* **507**, 462-470 (2014).

7. Akalin A*, et al.* methylKit: a comprehensive R package for the analysis of genome-wide DNA methylation profiles. *Genome biology* **13**, R87 (2012).

8. Akalin A*, et al.* Base-pair resolution DNA methylation sequencing reveals profoundly divergent epigenetic landscapes in acute myeloid leukemia. *PLoS genetics* **8**, e1002781 (2012).

9. Kandoth C*, et al.* Mutational landscape and significance across 12 major cancer types. *Nature* **502**, 333-339 (2013).

10. Cancer Genome Atlas Research N. Integrated genomic characterization of papillary thyroid carcinoma. *Cell* **159**, 676-690 (2014).

11. Cancer Genome Atlas N. Comprehensive molecular portraits of human breast tumours. *Nature* **490**, 61-70 (2012).

12. Assenov Y, Muller F, Lutsik P, Walter J, Lengauer T, Bock C. Comprehensive analysis of DNA methylation data with RnBeads. *Nature methods* **11**, 1138-1140 (2014).

13. Park C, Yu N, Choi I, Kim W, Lee S. lncRNAtor: a comprehensive resource for functional investigation of long non-coding RNAs. *Bioinformatics* **30**, 2480-2485 (2014).

14. Iyer MK*, et al.* The landscape of long noncoding RNAs in the human transcriptome. *Nature genetics* **47**, 199-208 (2015).

15. Hernandez-Ferrer C, Quintela Garcia I, Danielski K, Carracedo A, Perez-Jurado LA, Gonzalez JR. affy2sv: an R package to pre-process Affymetrix CytoScan HD and 750K arrays for SNP, CNV, inversion and mosaicism calling. *BMC bioinformatics* **16**, 167 (2015).

16. Van Loo P*, et al.* Allele-specific copy number analysis of tumors. *Proceedings of the National Academy of Sciences of the United States of America* **107**, 16910-16915 (2010).

17. Watkins J*, et al.* Genomic Complexity Profiling Reveals That HORMAD1 Overexpression Contributes to Homologous Recombination Deficiency in Triple-Negative Breast Cancers. *Cancer discovery* **5**, 488-505 (2015).
